# From Cell Differentiation to Cell Collectives: *Bacillus subtilis* Uses Division of Labor to Migrate

**DOI:** 10.1371/journal.pbio.1002141

**Published:** 2015-04-20

**Authors:** Jordi van Gestel, Hera Vlamakis, Roberto Kolter

**Affiliations:** 1 Department of Microbiology and Immunobiology, Harvard Medical School, Boston, Massachusetts, United States of America; 2 Theoretical Biology Group, Groningen Institute for Evolutionary Life Sciences, University of Groningen, Groningen, The Netherlands; Massachusetts Institute of Technology, UNITED STATES

## Abstract

The organization of cells, emerging from cell–cell interactions, can give rise to collective properties. These properties are adaptive when together cells can face environmental challenges that they separately cannot. One particular challenge that is important for microorganisms is migration. In this study, we show how flagellum-independent migration is driven by the division of labor of two cell types that appear during *Bacillus subtilis* sliding motility. Cell collectives organize themselves into bundles (called “van Gogh bundles”) of tightly aligned cell chains that form filamentous loops at the colony edge. We show, by time-course microscopy, that these loops migrate by pushing themselves away from the colony. The formation of van Gogh bundles depends critically on the synergistic interaction of surfactin-producing and matrix-producing cells. We propose that surfactin-producing cells reduce the friction between cells and their substrate, thereby facilitating matrix-producing cells to form bundles. The folding properties of these bundles determine the rate of colony expansion. Our study illustrates how the simple organization of cells within a community can yield a strong ecological advantage. This is a key factor underlying the diverse origins of multicellularity.

## Abstract 2

In the bacterium *Bacillus subtilis*, cooperation between surfactin-producing and matrix-producing cells drives migration over solid surfaces by the formation of filamentous loops at the edge of the colony. The growth of filamentous loops and their folding properties determine the rate of migration.

## Author Summary

Some problems can be solved only when individuals act together. This applies to bacteria in the same way that it applies to humans. Here we study how bacteria overcome the environmental challenge of migration over a solid surface by bundling their forces. Migration can be a significant environmental challenge for bacteria, especially when food sources are distributed far apart and have to be reached by movement along a solid surface, where swimming motility does not work. We show that *Bacillus subtilis*—a common inhabitant of the soil—migrates over a solid surface by forming multicellular structures. Migration depends on the synergistic interaction of two cell types: surfactin-producing and matrix-producing cells. Surfactin-producing cells facilitate migration by reducing the friction between cells and their substrate, thereby allowing matrix-producing cells to organize themselves into bundles that form filamentous loops at the colony edge. Using time-course microscopy, we observe that the filamentous loops drive migration by pushing themselves away from the colony. A mathematical model further shows that the folding properties of these loops are critical for the rate of colony expansion. Thus, not only do cells act together to overcome the challenge of migration, they also divide labor, in that different cell types specialize on distinct tasks.

## Introduction

Many properties of biological systems come about through the interactions of the parts that compose such systems. These so-called collective properties are said to “emerge” from these interactions, because they cannot be produced by the parts separately [1–3]. The most remarkable collective properties are found in multicellular organisms, where cell–cell interactions result in a bewildering diversity of forms and functions that cannot be generated by the cells in isolation [4–7]. Cell differentiation is an important factor underlying this diversity [8,9]. Cell types that differ in their adhesive properties, motility, or shape interact with each other and thereby guide developmental change [5,10]. When a collective property is adaptive, cell types that give rise to this property can be favored by selection [11–13]. The evolution of cell differentiation and collective properties can therefore go hand in hand [5].

Collective properties are often studied in species where cells can live independently, but often choose not to. These species are ideal for studying why and when cells form collectives and how these collectives come about. One of the most remarkable examples of such voluntary cell collectives comes from the soil-dwelling bacterium *Myxoccocus xanthus* [3,14]. During predation of other bacteria, thousands of *M*. *xanthus* cells coordinate their behavior to lyse and degrade prey [15]. When nutrient levels decrease, *M*. *xanthus* cells aggregate and assemble into a fruiting body filled with many thousands of spores [16,17]. The aerial projections of the fruiting body are thought to aid in spore dispersal [18]. Whereas it is a major challenge for individual cells to disperse, the cell collectives solve this problem by sticking out from the soil [1,2,8,9,19]. Dispersal is a major challenge for many soil-dwelling microorganisms. As a result, aerial spore-containing structures evolved independently in a number of bacterial and eukaryotic species, through the process of convergent evolution [20–22].

Another major challenge for soil-dwelling organisms is migration: how to get from one soil particle to the next. Without the possibility of swimming through liquid, cells have to find alternative ways to migrate [9]. These are often studied by examining colony growth patterns [19,23–27]. For example, *Paenibacillus vortex* migrates by making vortices that consist of millions of cells that swirl around over agar surfaces, producing beautiful fractal growth patterns [19,28,29]. A closely related species, *Bacillus mycoides*, forms chiral branching patterns that orient clockwise or counterclockwise while expanding over the agar surface [30]. A number of other species from the same bacterial families, Bacillaceae and Paenibacillaceae, have been studied as well with respect to colony growth patterns [26,31–35]. In all cases, cells solve the challenge of migration by migrating together. Yet how cells coordinate migration is often unknown: which cell types drive migration and how do they interact? This lack of knowledge is partly because little is known about the cell types that are expressed during colony growth. Interestingly, one species from the Bacillaceae family, *B*. *subtilis*, produces a number of different cell types and has been intensely studied with respect to cell differentiation [36]. The phenotypes of these cell types are well characterized [37]. *B*. *subtilis* therefore is the ideal species to examine if and how different cell types guide the migration of cell collectives. Furthermore, it gives a unique opportunity to examine how adaptations at the cell level relate to the collective properties that emerge from them.

*B*. *subtilis* can express at least five distinct cell types, which are often studied in the context of biofilm formation. Each of these cell types is associated with a unique set of phenotypes: motility, surfactin production, matrix production, protease production, and sporulation [36–39]. Motile cells synthesize flagella that can be used for swimming. Surfactin-producing cells secrete surfactin, a surfactant that reduces water surface tension [21,40], functions as a communication signal [41,42], and acts as an antimicrobial [43]. Matrix-producing cells secrete an extracellular polysaccharide (EPS) and the structural protein TasA [44,45]. EPS acts as a “glue” that surrounds cells inhabiting the biofilm. In addition, colony wrinkling requires EPS, and under some conditions, colony expansion also depends on EPS [46–48]. TasA assembles into amyloid-like fibers that attach to the cell wall and, like EPS, is required for colony wrinkling [45,49,50]. Since *tasA* and *eps* mutants complement each other when cocultured, TasA and EPS are considered common goods that are shared between cells [45,51]. In addition to EPS and TasA, matrix-producing cells secrete antimicrobial compounds that can kill sibling cells and other soil-dwelling organisms [52]. Protease-producing cells secrete proteases that facilitate nutrient acquisition [53,54]. Finally, cells can differentiate into spores: stress-resistant cells that can survive long periods of desiccation and nutrient limitation [55]. The regulatory mechanisms underlying cell differentiation in *B*. *subtilis* are well-characterized [37]. In addition, most cell types have been associated with some colony-level properties, although a detailed causal relation is often lacking [56].

Here we study how cell differentiation affects the migration of cell collectives during *B*. *subtilis* colony expansion via sliding motility. We grow bacteria on a specific medium that prevents cells from swimming and swarming (which both rely on flagella), but still allows for colony expansion. In this way, we can examine whether colony expansion depends on cell differentiation, and if so, how the interactions between cell types drive migration. We show that migration depends critically on two cell types: surfactin-producing and matrix-producing cells. Together they drive migration through a mechanism in which cell collectives form highly organized bundles at the colony edge, which we have termed “van Gogh bundles.” Van Gogh bundles are formed from many tightly aligned filaments consisting of chains of cells. They appear elastic and fold into filamentous loops that push themselves away from the colony. Surfactin-producing and matrix-producing cells divide labor during the formation of van Gogh bundles. We propose that surfactin-producing cells reduce the friction between cells and their substrate, which facilitates formation of the van Gogh bundles by the matrix-producing cells. Whereas EPS production is necessary for the formation of these bundles, TasA seems to fine-tune their biophysical properties. Finally, as a complement to the experiments, a mathematical model illustrates how simple cellular properties can affect a bundle’s folding properties and hence the migration rate.

## Results

### Cell Types That Control Colony Expansion

**Fig 1:**
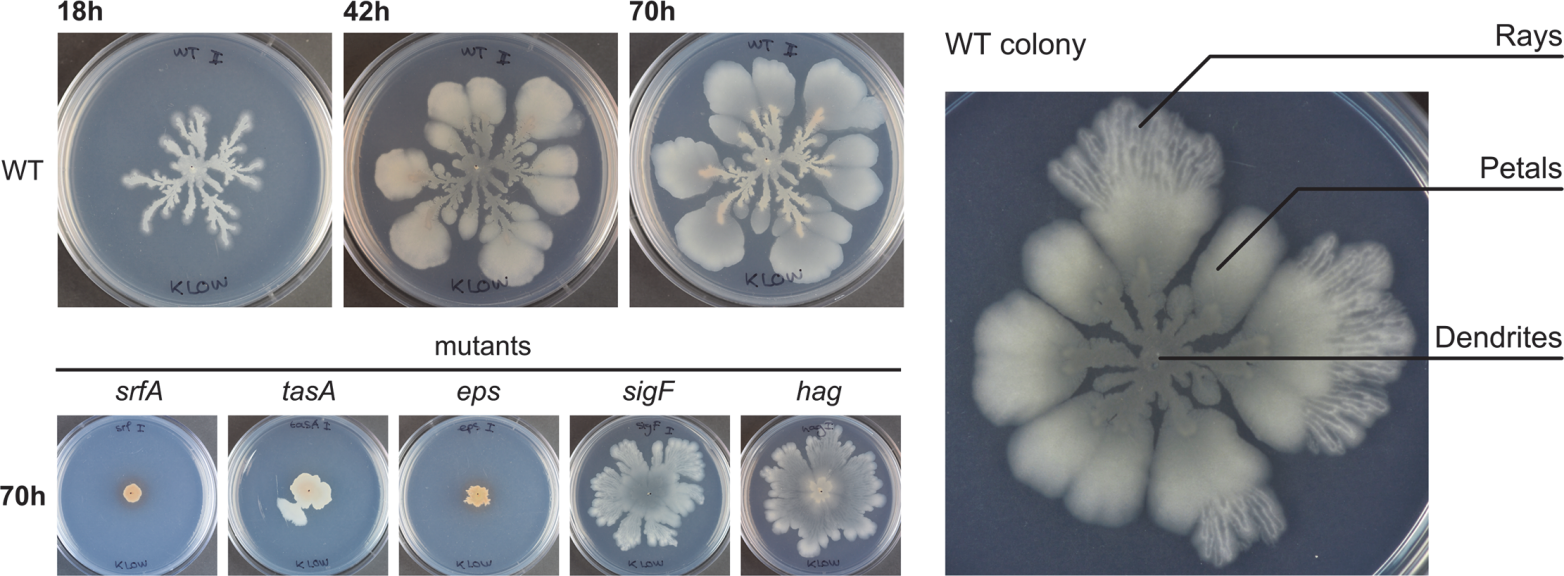
Colony expansion in wild type and biofilm-related mutants. Left: time course experiment of colony growth in WT and colony expansion in *srfA*, *tasA*, *eps*, *sigF*, and *hag* mutants, which are defective in producing surfactin, TasA, EPS, sporulation, and motility, respectively. Colonies are toothpick inoculated onto MSggN medium as described in the Materials and Methods. Right: WT colony after 70 h. The different regions of the colony are named: dendrites, petals, and rays.

We studied migration by examining colony growth on MSggN [57]. MSggN is a growth medium that induces colony expansion and resembles the biofilm-inducing medium, MSgg, that is typically used to study cell differentiation in the context of *B*. *subtilis* biofilms [21,38,57]. Colony expansion is more apparent on MSggN than on MSgg, which makes the former more suitable for studying migration (see Materials and Methods). Colony growth on MSggN consists of two main phases that are morphologically distinct (Fig 1; see also [57]). First, the colony forms dendrites that spread radially from the inoculum. Second, phenotypically distinct outgrowths, which we call “petals,” appear at the end of the dendrites. In some instances the petals change into another morphological structure at the colony edge, which we call “rays.” The distinct growth phases do not result from genetic change, because cells from the morphologically distinct regions of the colony behave the same as wild type (WT) when re-inoculated onto a fresh growth medium (S1 Fig).

To determine which cell types are involved in colony expansion we tested mutants deficient in the production of surfactin (*srfA* mutant), extracellular matrix (*eps* and *tasA* mutants), spores (*sigF* mutant), and flagella (*hag* mutant). Both surfactin-producing and matrix-producing cells were necessary for colony expansion, whereas motility and sporulation mutants showed a nearly WT colony expansion (Fig 1). We examined two matrix-related mutants: *eps* and *tasA*. While the *eps* mutant did not show any degree of colony expansion, *tasA* mutants did expand beyond the colony boundaries present at inoculation, although the expansion was much less than that of WT (Fig 1). These results are in agreement with previous studies that showed that *B*. *subtilis* colony expansion on MSggN is independent of flagellum formation, but requires surfactin production [57–59]. In addition, our experiment showed that matrix-producing cells are also required for colony expansion.

### Colony Expansion in Chimeric Colonies

**Fig 2:**
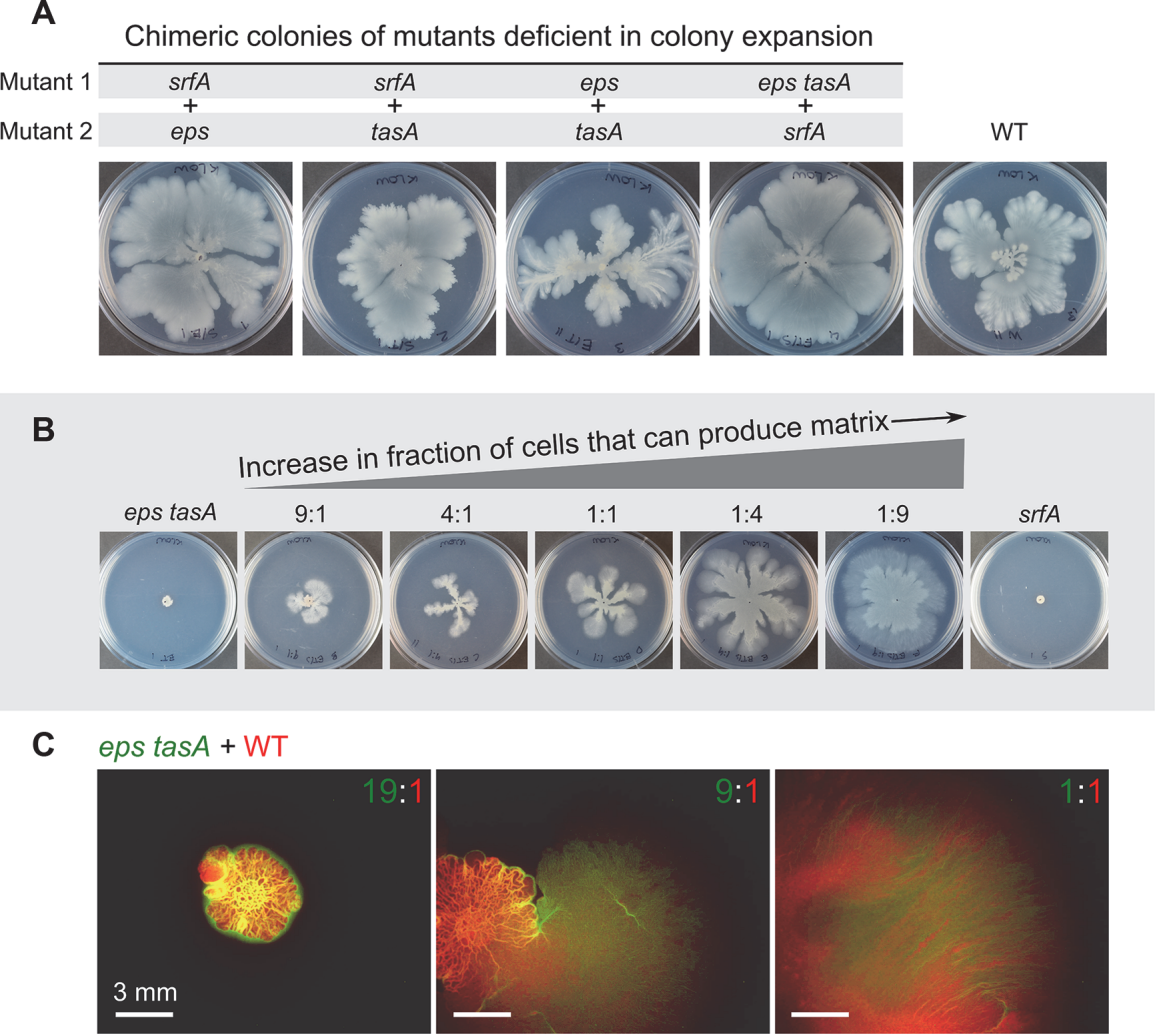
Colony expansion in chimeric colonies of sliding-deficient mutants. (A) Chimeric colonies of different pairwise combinations of sliding-deficient mutants (imaged 52 h after inoculation). Strains were mixed 1:1, and 2 μl of the mix was spotted in the center of the plate. (B) Colony expansion of *eps tasA* + *srfA* chimeras when inoculated in different ratios of *eps tasA*:*srfA* (imaged after 24 h): 9:1, 4:1, 1:1, 1:4, and 1:9. (C) Colony expansion of *eps tasA*–YFP (false-colored green) + WT-mKate2 (colored red; see S1 Table for specifications) chimeras for different initial ratios of *eps tasA*:WT (imaged after 32 h): 19:1, 9:1, and 1:1. Yellowish regions in the colony correspond to colony parts where both *eps tasA* and WT cells occur. In the rightmost fluorescence image, only the colony edge is shown, as the colony was too big for a single microscopy image (the center of this colony is towards the lower left corner). Images were taken with a stereomicroscope.

To examine whether colony expansion could be recovered by extracellular complementation, different pairs of expansion-deficient mutants were cocultured as chimeric colonies [60]. Such two-mutant cocultures can reveal something about the interactions between different cell types during colony growth [45,61]. All examined chimeric colonies in which mutant cells were mixed at a 1:1 ratio showed a partial to full recovery of colony expansion when compared to the WT (Fig 2A). Interestingly, two of the chimeric colonies appeared to outperform WT in the extent of colony expansion: *srfA* + *eps* and *eps tasA* + *srfA*. Thus, the task differentiation of matrix and surfactin production by mutant strains enhanced the degree of colony expansion.

To further examine these fast-expanding chimeric colonies, we varied the initial ratio of strains deficient in surfactin (*srfA*) and matrix (double mutant *eps tasA*) production. Colonies were compared 24 h after inoculation. In chimeric colonies that contained many *eps tasA* mutant cells, there was little colony expansion (Fig 2B, 9:1 *eps tasA*:*srfA*), and in chimeric colonies with many *srfA* mutant cells, colonies expanded far (Fig 2B, 1:9 *eps tasA*:*srfA*). In the *eps tasA* + *srfA* chimera, *eps tasA* mutant cells are responsible for surfactin production and *srfA* mutant cells are responsible for matrix production. Therefore, we conclude that the extent of colony expansion is mostly constrained by the number of cells that produce matrix: a small number of surfactin-producing cells is sufficient to fully restore colony expansion (see Fig 2B, 1:9 *eps tasA*:*srfA*), while a small number of matrix-producing cells is not (see Fig 2B, 9:1 *eps tasA*:*srfA*).

Finally, we examined chimeric colonies of strains that were marked with different fluorescent reporters. This allowed us to determine how strains mixed in space when grown together. Interestingly, not all strain combinations mixed homogeneously. When strains differed in terms of matrix production, for example, in a chimera of a matrix-deficient mutant (*eps tasA*) and the WT strain, spatial segregation was observed (Fig 2C). This directly affected colony expansion. Even though the WT strain has the potential to fully expand over the agar plate, it could not expand when strongly outnumbered by the matrix-deficient strain in the initial inoculum (Fig 2C, 19:1 *eps tasA*:WT). This suggests that matrix-deficient cells prevent WT cells from migrating. Mutant cells might simply block WT cells by surrounding them at the colony edge (Fig 2C). Alternatively, in the presence of mutant cells, the appropriate environmental signals to trigger colony expansion might be lacking. When the fraction of WT cells in the inoculum increased (from left to right in Fig 2C), the WT could expand over the agar plate. In that case, both strains were found in the expanded section of the colony. Thus, in addition to matrix-producing cells facilitating colony expansion, matrix-deficient cells can inhibit colony expansion.

### Temporal Expression Pattern of Surfactin-Producing and Matrix-Producing Cells

The chimeric colonies showed that colony expansion depends on the presence of both surfactin-producing and matrix-producing cells. In the next sections we examine how these cell types interact in the WT and consequently drive migration. To study surfactin-producing and matrix-producing cells in a WT strain, we used a double-labeled strain in which the expression of two fluorescent reporters, genes coding for yellow (YFP) and cyan (CFP) fluorescent proteins, is under the control of the promoter for surfactin biosynthesis genes (P_*srfA*_) and the *tasA* operon promoter (P_*tapA*_), respectively [42]. Thus, in the double-labeled strain, surfactin-producing cells express YFP, and matrix-producing cells express CFP.

First, we examined the temporal gene expression dynamics by performing a time-course experiment. Colonies were examined every 2 h for 12 h after inoculation, as well as at 24 h and 31 h after inoculation (Materials and Methods). Since the *srfA* promoter is very weakly expressed, it was impossible to detect using flow cytometry. Instead, direct microscopy was performed on the colony samples, which were first dispersed in phosphate buffered saline (PBS) buffer to get a representative fraction of cells. At every time point, microscopy pictures were taken from a labeled WT strain (*n* = 20–50 microscopy images) and, as a control, an unlabeled WT strain (*n* = 10–30 microscopy images). Since it was impossible to accurately analyze all of the images manually (*n* = 439), a MatLab program was used to quickly select, process, and statistically analyze the images (see Materials and Methods for details; [62]).

**Fig 3:**
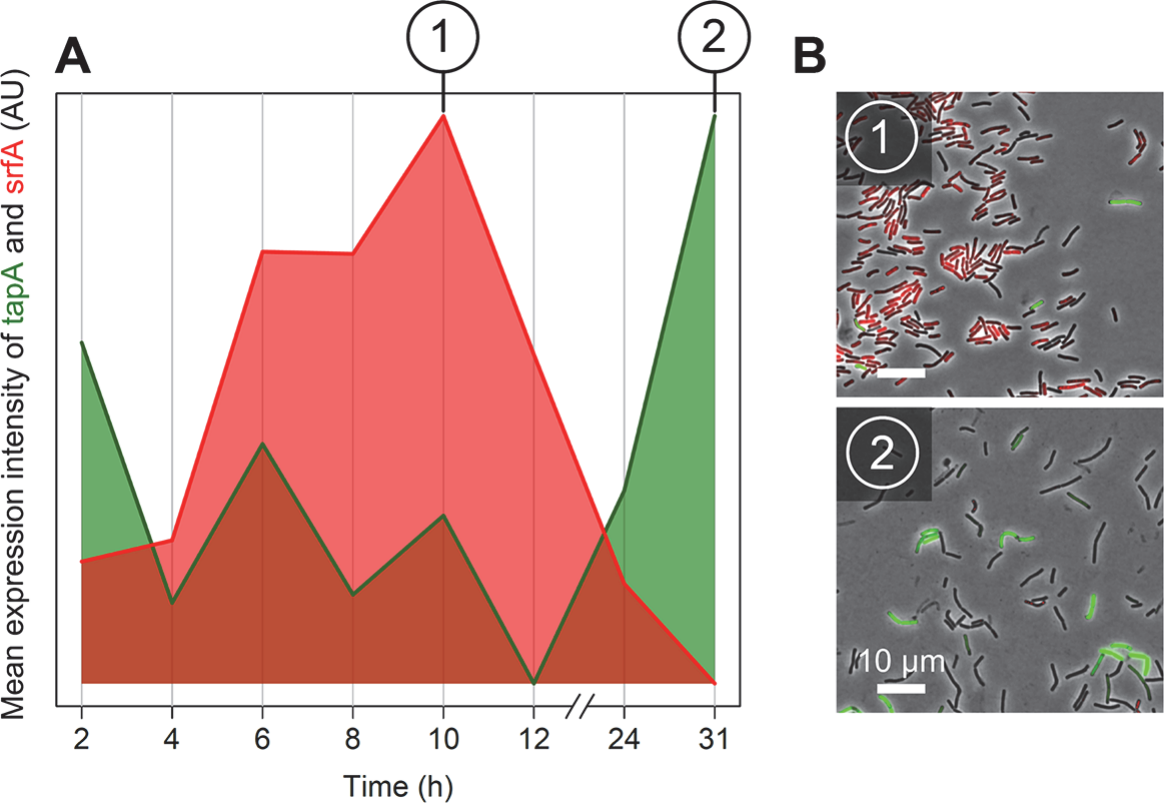
Temporal gene expression dynamics of *srfA* and *tapA* during colony expansion in wild-type cells. Surfactin- and matrix-producing cells are monitored in a WT strain harboring promoter fusions (P_*srfA*_-YFP and P_*tapA*_-CFP) of *srfA* and *tapA* to genes encoding yellow and cyan fluorescent proteins, respectively. (A) The average expression level of *tapA* and *srfA* was measured by microscopy 2, 4, 6, 8, 10, 12, 24, 31 h after inoculation. The average expression level is equal to the average fluorescence intensity in labeled WT cells (*n* = 20–50 microscopy images per time step) minus that in non-labeled WT cells (*n* = 10–30 microscopy images per time step). Fluorescence intensity data were acquired from segmented microscopy images (*n* = 439; containing many thousands of cells). (B) Representative microscopy images from colonies dissected at 10 h (1) and 31 h (2) after inoculation. AU, arbitrary units. Cells expressing *srfA* are false-colored red, and cells expressing tapA are false-colored green.

Fig 3A shows the expression of *srfA* and *tapA* over time. The expression pattern is characterized by two phases: in the first phase there is a peak in the average expression of *srfA*, while in the second phase there is sharp increase in the average expression of *tapA* (Fig 3A). Fig 3B shows a representative image from each of these two phases. At the onset of colony growth there is also a slight peak in *tapA* expression, which is due to background expression in the inoculation conditions (for details see Materials and Methods). When taking the time frame of gene expression into consideration, the up-regulation of *srfA* corresponds to dendrite formation, and the up-regulation of *tapA* corresponds to petal formation (Figs 1 and 3). The distinct growth phases that are apparent at the macroscopic level therefore relate to gene expression dynamics at the cell level (microscopic). The same microscopy images were used to examine the co-expression of *srfA* and *tapA*. As expected from previous studies [42], the expression of *srfA* and the expression of *tapA* were mutually exclusive (S1 Text; S2 Fig). This confirmed that also for our growth conditions, surfactin-producing and matrix-producing cells are mutually exclusive and distinct cell types (S2 Fig).

### Spatial Expression Pattern of Surfactin-Producing and Matrix-Producing Cells

Next we studied the spatial arrangement of surfactin-producing and matrix-producing cells. Colonies were examined by cutting a piece of the agar at the colony edge. This agar piece was subsequently flipped onto a glass-bottom well, sandwiching the cells between the coverslip and an agar pad, and the cut piece of colony edge was subjected to a detailed microscopic examination (for details see Materials and Methods). The advantage of this technique is that intact cell collectives could be observed, as they would occur in growing colonies. Examining these cell collectives is particularly important because it might help in understanding how cells migrate during colony expansion. However, a disadvantage of the technique is that colonies can be examined only at the edge, where a monolayer of cells exists, which is necessary for accurate quantification of fluorescent images.

**Fig 4:**
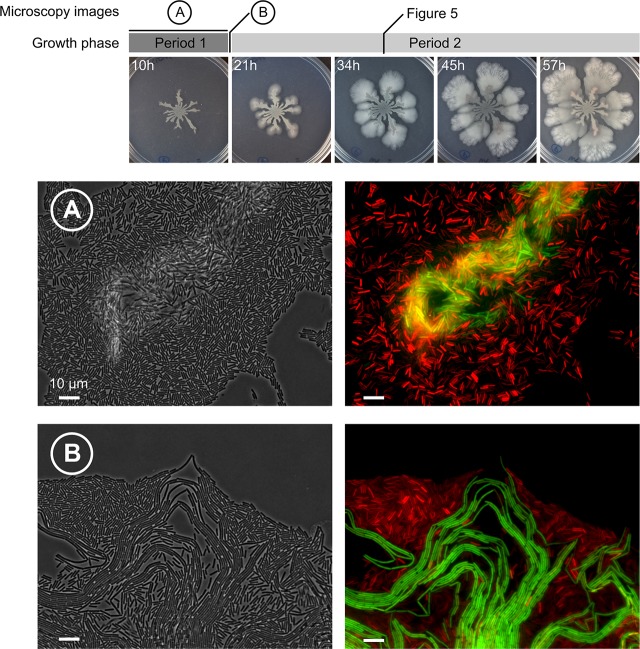
Spatial expression pattern of *srfA* and *tapA* during colony expansion in wild-type cells. Top: colony growth 10, 21, 34, 45, and 57 h after inoculation. Colony expansion is divided in two growth periods: (1) dendrite formation (<11–13 h) and (2) petal-shaped colony outgrows (>11–13 h). (A) Microscopy image of a cellular aggregate that appeared in the first growth period. Red and green fluorescent cells represent, respectively, surfactin- and matrix-producing cells in the double-labeled P_*tapA*_-CFP P_*srfA*_-YFP WT strain (CFP and YFP are artificially colored green and red, respectively). (B) Cellular aggregate at the transition from the first to the second growth period. All microscopy images were made at the edge of the colony with an inverted microscope. Cellular aggregates were examined in colonies inoculated by toothpick or pipet and from a passaging experiment (see Materials and Methods). The observed clumping was qualitatively the same for all inoculation conditions.

The colony edge was dissected at different time points ranging over two colony growth phases: dendrite formation (<11–13 h) and petal formation (>11–13 h) (summarized at the top of Fig 4). During dendrite formation, cells aggregate into clumps. These clumps consist of matrix-producing cells (false-colored green) and are surrounded by surfactin-producing cells (false-colored red, Fig 4A). The clumps appear within a few hours after inoculation. Even when we made certain that there were no surfactin-producing or matrix-producing cells present in the inoculum (by performing a passaging experiment, see Materials and Methods) clumps formed rapidly. The clumps were relatively unorganized: the shape, size, and location varied strongly (Fig 4A shows one example). Although both surfactin producers and matrix producers are necessary for dendrite formation, as is evident from the mutant phenotypes (Fig 1), it is unclear if and how these clumps contribute to dendrite formation.

**Fig 5:**
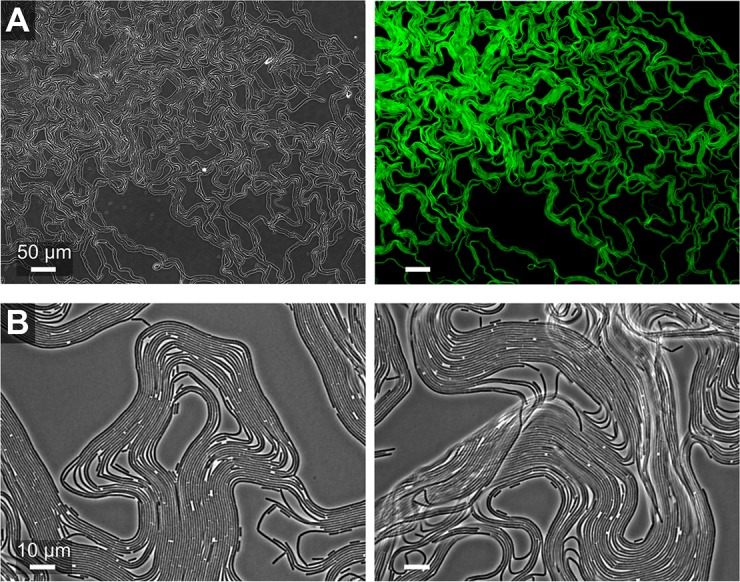
Van Gogh bundles and *tapA* expression at the colony edge 34 h after inoculation. (A) Composite image of van Gogh bundles at the colony edge, consisting of multiple microscopy frames. Left: phase-contrast image. Right: green cells represent matrix-producing cells (i.e., *tapA* expression) in the double-labeled P_*tapA*_-CFP P_*srfA*_-YFP WT strain (CFP is artificially colored green). *srfA* expression is not shown in the composite image because of bleaching problems with multi-image acquisition. (B) Phase-contrast microscopy images of van Gogh bundles at high magnification.

During the transition between the first growth phase (dendrite growth) and the second growth phase (formation of petal-shaped colony outgrowths at the tip of the dendrites) (Fig 1), a new type of aggregate appeared (Fig 4B). As was observed for clumps, there was strong spatial segregation between surfactin-producing and matrix-producing cells: matrix-producing cells occurred inside the bundle, whereas surfactin was expressed by cells surrounding the bundle. Interestingly, in contrast to aggregates in the first growth phase, the bundles appear organized. The bundles consist of many cellular filaments that are arranged side by side and are only a single cell layer thick. During the transition, the bundles seem to push themselves out of the colony edge (i.e., away from the single cells). The coordinated appearance of the bundles is even more striking at later time points. Fig 5A and 5B show the colony edge after 34 h of colony growth. At this point, the colony edge consists of only the well-organized bundles. Henceforth, we refer to these bundles as “van Gogh bundles,” because of the resemblance of these cell collectives to the brushstrokes in van Gogh’s *The Starry Night*.

The organized appearance of van Gogh bundles results from a remarkably strong alignment of cells inside the bundles (S2 Text). This is especially apparent when comparing the alignment of cells inside van Gogh bundles to the alignment of cells at the colony edge earlier in colony growth (S3–S5 and S7 Figs). In fact, when considering only the alignment of cells, one can discriminate regions in a microscopy image that contain van Gogh bundles from regions that do not (S6 Fig). Furthermore, van Gogh bundles appear flexible. When flipping the colony onto the glass-bottom well, the bundles sometimes folded (Fig 5B), yet they hardly ever broke. Thus, adhesive forces between the cells must keep them aligned and attached such that shear forces or friction do not break them.

### Van Gogh Bundles: EPS and TasA

In the chimeric colonies of strains with expansion-deficient mutations, described above, colony expansion was partly or fully recovered (Fig 2). From the previous section, one expects that the recovery of colony expansion results from the formation of van Gogh bundles. To test this, we examined the chimeric colonies using microscopy. Colonies were examined at the start of the second growth phase (i.e., the start of petal outgrowths), when both single cells and van Gogh bundles were expected to be present (see S8 Fig). The strains in the chimeric colonies were marked with fluorescent reporters, so that their spatial arrangement could be examined as well.

**Fig 6:**
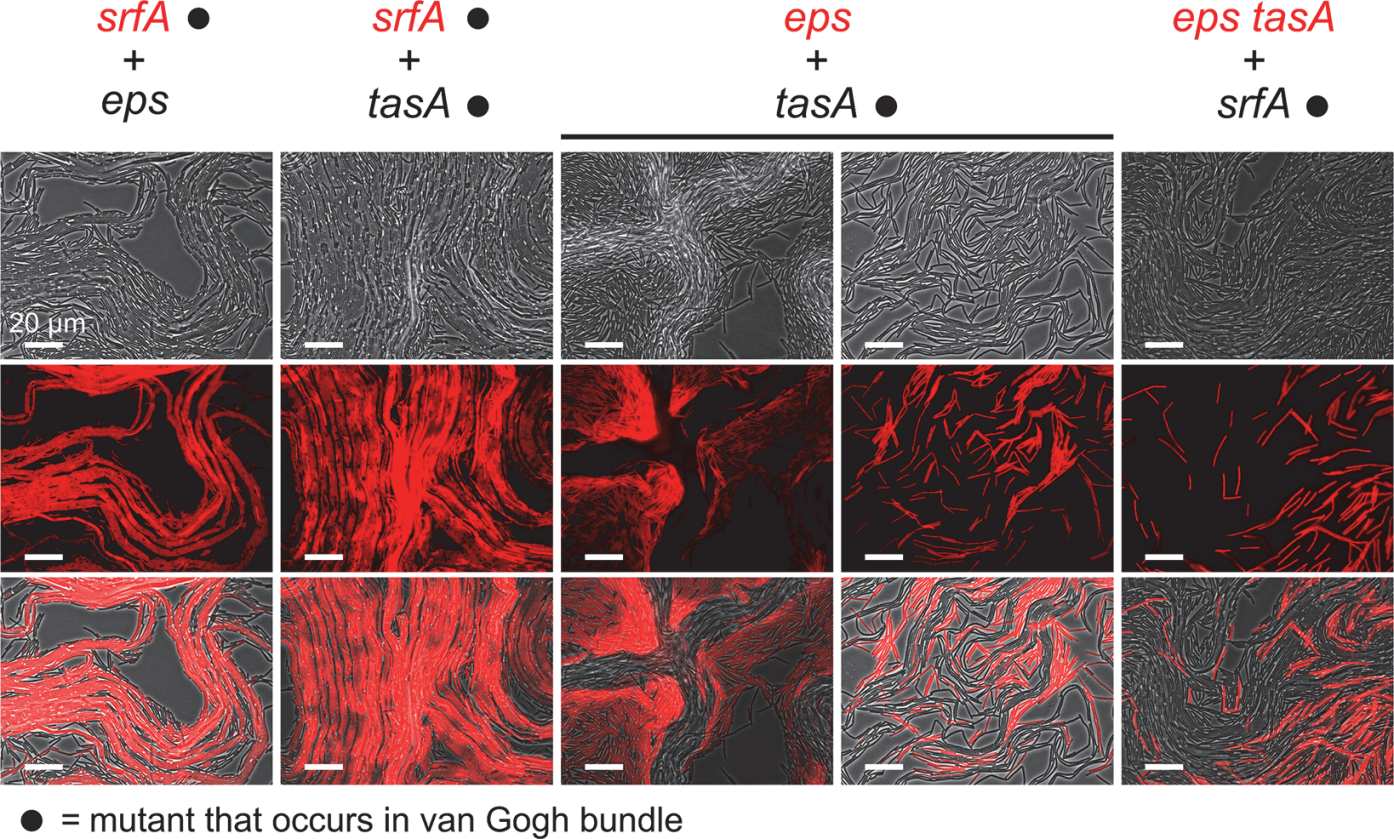
Van Gogh bundle formation in mutant chimeras. Microscopy images were taken from the colony edge 21 h after inoculation, which is close to the temporal transition from the first to the second growth period in mutant chimeras mixed at a 1:1 ratio. One strain per chimera is labeled with constitutive expression of mKate2 (false-colored red) as indicated below. The top, middle, and bottom rows of images show, respectively, the phase-contrast, fluorescence, and overlay microscopy images. Four mutant chimeras were examined (columns): (1) *srfA* (mKate2) + *eps*, (2) *srfA* (mKate2) + *tasA*, (3) *eps* (mKate2) + *tasA*, and (4) *eps tasA* (mKate2) + *srfA*. For the *eps*-mKate2 + *tasA* chimera, the two columns show images from regions without (left) and with (right) visible van Gogh bundles. The left image without van Gogh bundles, but with strong cell clumps, was acquired from a dendrite that had not made the transition to petal growth yet (such dendrites were not present for the other chimeras, because they developed more quickly; see S8 Fig).

Fig 6 shows that all chimeric colonies produced van Gogh bundles, although the bundles were not always as apparent as those in WT colonies (e.g., the *eps* + *tasA* chimera showed less apparent bundle formation). Thus, the recovery of colony expansion coincided with the emergence of van Gogh bundles during colony growth. In contrast, most mutants could not produce van Gogh bundles by themselves (see below). Interestingly, although both mutant strains were necessary for recovering the van Gogh bundles, not all cells became part of the van Gogh bundles. The fluorescent overlays show that the van Gogh bundles were made up of cells from the EPS-producing strains only. This is particularly apparent for the first and last mutant chimeras (e.g., *srfA* + *eps* and *eps tasA* + *srfA*), in which EPS-deficient cells never formed cell chains that were part of the van Gogh bundle (Fig 6). In cases where both strains produced EPS, such as in the *srfA* + *tasA* chimera, van Gogh bundles did consist of cells from both strains, with the cell chains inside the van Gogh bundles belonging to either one of them. All in all, these results indicate that EPS is strictly required for the formation of van Gogh bundles, presumably for the adhesion between neighboring cell chains.

**Fig 7:**
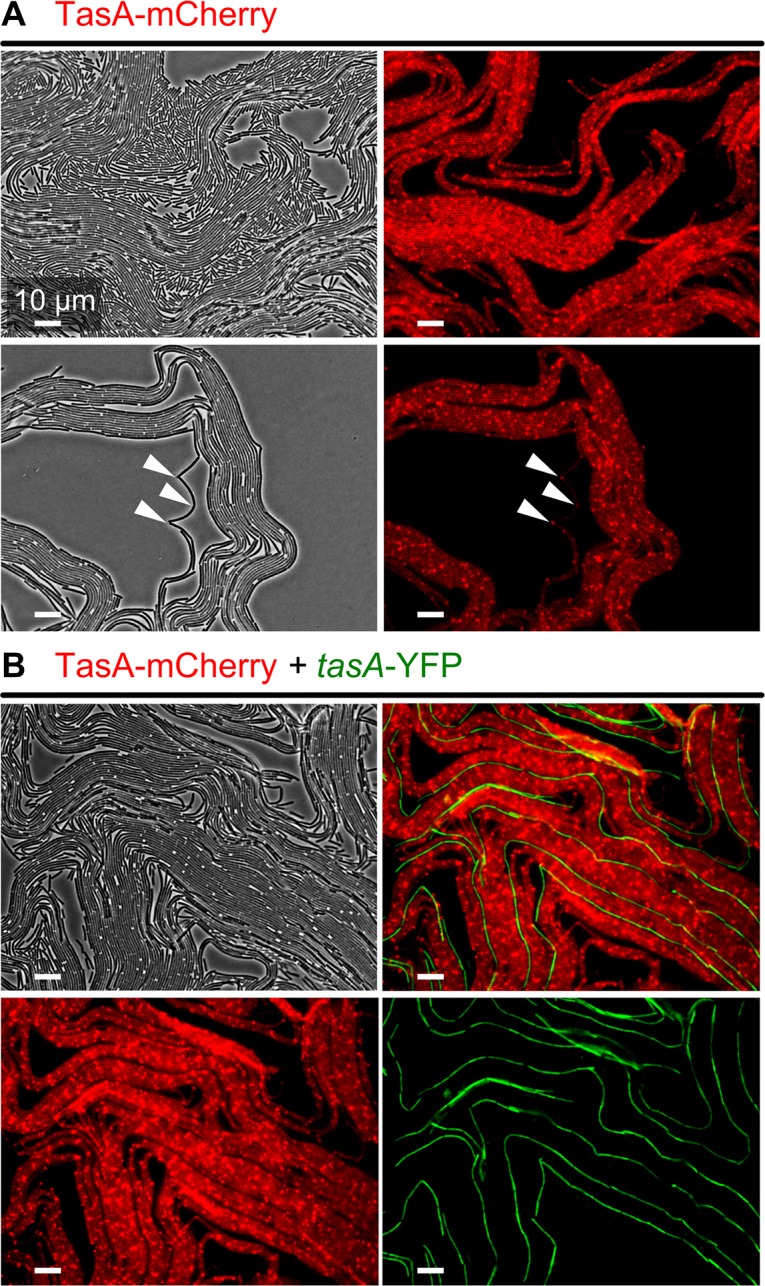
Localization of TasA protein in van Gogh bundles. (A) Representative microscopy images of the WT strain with a protein fusion of TasA to mCherry (TasA-mCherry) at the colony edge 20 h after inoculation (phase-contrast [left] and fluorescent [right] images). Red corresponds to localization of TasA protein. White arrowheads indicate illustrative points in the images that show TasA localization at the pole-to-pole interaction zone between cells. (B) Chimera of TasA-mCherry + *tasA* mutant at the colony edge 26 h after inoculation (i.e., in the second growth phase). The *tasA* mutant is labeled with a constitutively expressed YFP gene (false-colored green). Van Gogh bundles consist of both strains. Phase-contrast and fluorescent images are shown.

After evaluating the mutant chimeras, it is still unclear what the role of TasA is in the formation of van Gogh bundles. TasA was not strictly required for the formation of van Gogh bundles (see the *eps* + *tasA* chimera in Fig 6). Yet, the *tasA* mutant was partly impaired in colony expansion (Fig 1). In order to evaluate the role of TasA, we examined the distribution of TasA protein directly by using a fusion of TasA and a red fluorescent protein (the fusion protein is designated TasA-mCherry). TasA-mCherry was examined by microscopy during the transition from the first to the second growth period. Previous studies suggested that TasA is freely shared between cells in the colony, since *tasA* mutants could be complemented when grown together with TasA-producing cells [45,50]. Interestingly, Fig 7A shows that TasA was predominantly localized to the van Gogh bundles—where TasA is also produced—and only a limited fraction of TasA diffused to the surrounding single cells (see S3 Text and S9 Fig). In fact, TasA particularly localized to the “pole to pole” interactions between cells (see arrowheads in Figs 7A and S10). Thus, in contrast to previous studies, our results suggest that there is only limited diffusion of TasA.

To examine whether TasA is shared between neighboring cells inside the van Gogh bundle, we examined a chimeric colony of TasA-mCherry + *tasA* mutant. Since the strain producing the fusion TasA-mCherry and the *tasA* mutant strain can form van Gogh bundles together (i.e., they both produce EPS), this chimera allows us to examine whether TasA produced by the TasA-mCherry strain is shared with the *tasA* mutant cells inside the van Gogh bundle (Fig 7B). Indeed, a small fraction of TasA diffused from the TasA-producing cells to the *tasA* mutant cells (S11 Fig). However, interestingly, there was no accumulation of TasA at the pole-to-pole interactions between *tasA* mutant cells (Figs 7B and S11). Thus, TasA accumulated only at the cell poles of TasA-producing cells inside the van Gogh bundle. It is plausible that a large fraction of the TasA produced by a WT cell localizes to its own poles.

In summary, van Gogh bundles are cell collectives that consist solely of matrix-producing cells but that require the presence of surfactin producers for their development. This is further confirmed by the fact that colony expansion in *srfA* mutants can be recovered by adding surfactin exogenously (S12 Fig; [58]). The matrix-producing cells secrete EPS and TasA. While EPS is absolutely necessary for the formation of a van Gogh bundle, TasA seems to fine-tune the cell-to-cell interactions.

### Van Gogh Bundles: Migration

In the previous sections we showed that colony expansion coincides with the formation of van Gogh bundles, which are formed when both surfactin-producing and matrix-producing cells are present. Surfactin functions as a surfactant and facilitates colony expansion by reducing the friction between cells and their substrate [57–59]. The question, however, remains as to how the cell collectives that organize themselves in van Gogh bundles migrate in space. To address this question, we examined van Gogh bundles in more detail.

**Fig 8:**
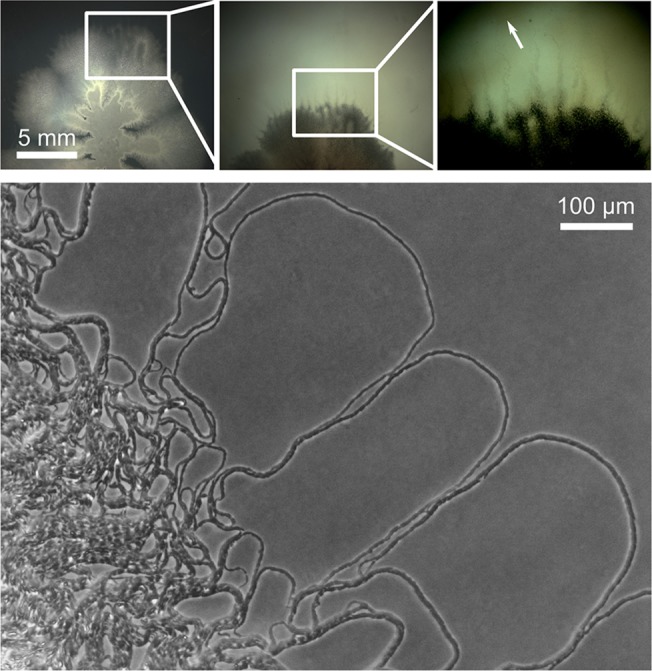
Van Gogh bundles and the emergence of filamentous loops at the colony edge. Images were taken at the colony edge of a WT strain grown for 28 h. The upper three images are insets of each other from a low (left) to high (right) magnification. The white arrow indicates the distance the van Gogh bundles spread over the agar plate. The lower image shows the van Gogh bundles at a higher magnification.

In order to analyze the structures that emerge at a larger spatial scale, we next imaged the van Gogh bundles at a lower magnification using a stereomicroscope. By using the stereomicroscope, no further manipulation of the colony was required, and growing colonies could be examined multiple times as growth progressed (the air objective does not disrupt the colony). Surprisingly, at lower magnification it became apparent that van Gogh bundles form large filamentous loops at the edge of the colony (Fig 8). These loops extend up to a few millimeters in length. We hypothesized that the van Gogh bundles migrate by simply pushing themselves away from the colony center as the filamentous loops grow. A time-lapse movie indeed confirmed our expectation (S1 Movie). Thus, colony expansion indeed emerges from the interaction of cells that organize themselves into van Gogh bundles.

**Fig 9:**
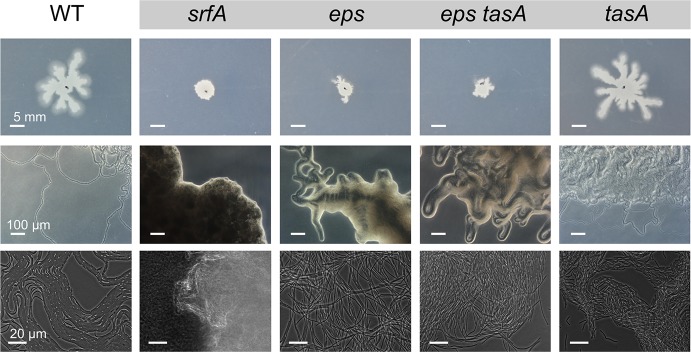
Van Gogh bundles and colony edge for different mutants. The upper row of images shows colonies 18 h after inoculation. The middle row of images shows the presence or absence of loops at the colony edge. The lower row of images shows the presence or absence of van Gogh bundles at the colony edge. In total, five strains were examined, WT and four sliding-deficient mutants: *srfA*, *eps*, *eps tasA*, and *tasA*. Scale bars are identical for all images in a row.

The lack of colony expansion in sliding-deficient mutants, with the exception of *tasA*, can be explained by the lack of van Gogh bundles and the associated loops at the colony edge (Fig 9). Interestingly, *eps* and *eps tasA* mutants do show chains of cells, similar to the chains of cells in van Gogh bundles, but they are not aligned with each other (Fig 9). The *tasA* mutant strain is mainly deficient in colony expansion during the second growth phase, as it can form dendrites (Fig 9). Furthermore, the filamentous loops at the edge of the *tasA* colony are typically smaller and show more folds than those of the WT (S13 Fig). We hypothesize that TasA, although not strictly required for the formation of van Gogh bundles, may fine-tune the folding properties of the bundles. This hypothesis is supported by the fact that TasA localizes to the pole-to-pole contact points between cells in the van Gogh bundles, where it potentially affects biophysical properties such as the bending rigidity (Fig 7). Interestingly, while the lack of TasA reduces colony expansion, the artificial overproduction of TasA does not enhance colony expansion (S14 Fig).

### Model of Filamentous Growth and Folding

Inspired by the folding differences between filamentous loops produced by the WT and those produced by *tasA* (S13 Fig), we wondered if and how cell-level properties (e.g., phenotype of a cell or cell–cell interactions) could affect the collective properties that we observed at the colony level. For this purpose, we constructed a simple phenomenological model. This model was not designed with the aim of quantitatively reproducing our experimental results, which at present is impossible given our limited knowledge of the biophysical properties of the van Gogh bundles. Rather, we aimed to illustrate how local cell interactions could shape colony-level properties. Previous models on multicellular development have shown that—through self-organization—simple cell-to-cell interactions can underlie complex properties that emerge at the organismal level [6,7,63]. Mathematical models are therefore a valuable tool to shape our intuition on the cell-level properties that are important for the qualitative patterns we observe at the colony level [4,7].

Inspired by models on epithelium folding [64–66], we modeled filaments of pole-to-pole-attached cells that grow in time (we ignored side-to-side attachment for simplicity). The model does not include the origin of filament formation, but instead examines filament growth. At every time step, cells can undergo one of three events: cell elongation, division, or turning (see Materials and Methods for modeling details). Cell elongation occurs with a certain growth rate, taken from a uniform distribution, and can result in cell division when the cell length exceeds a certain threshold (i.e., the maximum cell length); in that case the mother cell divides into two equally long daughter cells. Cells can also turn and change their spatial orientation with respect to their neighbors. Cells turn only when the new orientation—determined by a random change in a cell’s angle with respect to its neighbors—is energetically favored compared to the cell’s original orientation. In the energetically preferred position, a cell is perfectly aligned with its neighbors (i.e., there is no angle between two neighboring cells). The chance that a cell turns depends on the bending rigidity (see Materials and Methods). Cell elongation, division, and turning are local events that do not alter the spatial configuration of cells in other parts of the filament. Thus, the properties of the filament as a whole come about through the accumulation of local events.

**Fig 10:**
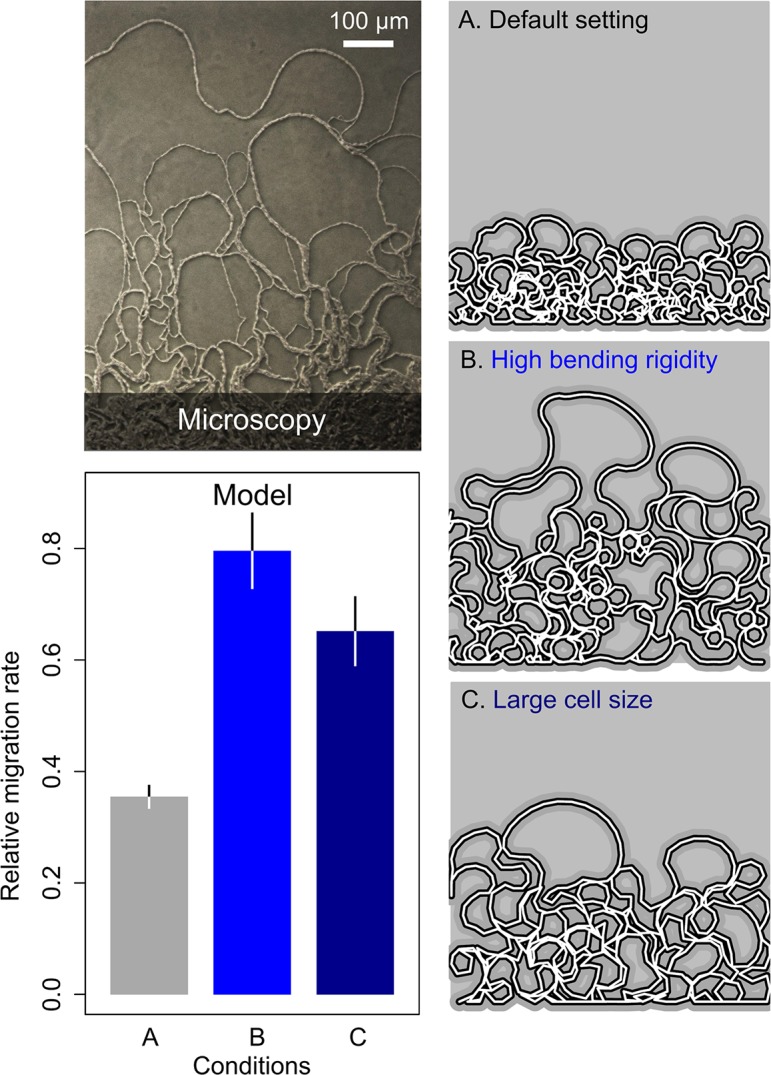
Model of filament growth and migration. Upper left: microscopy image of WT cells on the edge of the colony taken 18 h after inoculation. Lower left: relative migration rate for three parameter settings: (A) default parameter setting (grey), (B) high bending rigidity between cells (blue), and (C) large cell size (dark blue). Histograms and error bars show, respectively, mean and standard deviation (*n* = 10) in the extent of migration along the *y*-axis of the two-dimensional space (see images on the right; filament growth is initiated on the bottom). Right: filamentous loops at the end of the simulation for three representative runs, one for each of the three different parameter settings. See Materials and Methods for detailed model description and exact parameter settings (S2 Table).

As shown in Fig 10, these three simple cell-level behaviors are sufficient to produce expanding filamentous loops at the colony edge that look surprisingly similar to those observed in our experiments. Cell elongation and division result in undulations of the filaments (i.e., regions where the filaments bend slightly inwards or outwards). These undulations get smoothened as long as neighboring cells resist bending by strongly aligning with respect to each other (i.e., bending rigidity). However, when growth continues, the filament gets compressed and undulations increase. As a consequence, the filament starts folding. The folds turn into loops, which expand in space. As observed in the experimental results (Figs 8–10), the model gives rise to bigger loops at the edge of the colony (Fig 10).

To examine how small changes at the cell level affect the expansion of filamentous loops, two modeling parameters were varied: the maximal cell length and the bending rigidity. These parameters correspond to properties that probably can be influenced by a cell. For example, we showed that cells inside van Gogh bundles are longer than their single-cell siblings (Figs 4, 5, and S7; two-sample *t-*test: *p* < 10^−16^, df = 184), which suggests that cells can alter the length at which they divide. In addition, we showed that van Gogh bundles show a particularly strong alignment (S2 Text), which seems to partly depend on TasA that accumulates at the pole-to-pole interactions (Figs 7, 9, S10, and S13). This indicates that cells can alter their bending rigidity with respect to neighboring cells. Interestingly, in the model, both longer cells and higher bending rigidities result in filaments that fold less (Fig 10, conditions B and C). Longer cells reduce folding because there are fewer pole-to-pole interactions at which the filament could accumulate undulations. Likewise, when the bending rigidity is high, cells align more strongly, which results in less folding as well. The reduced tendency to fold increases the migration rate (Fig 10, compare conditions A, B, and C).

Our phenomenological model thus illustrates how small changes at the cell level can shape the collective properties that emerge at the colony level. The collective properties we examined are the expanding filamentous loops that appear at the colony edge. One can imagine that evolution favors adaptations at the cell level, like a strong cell-to-cell alignment, that result in a higher migration rate of the filamentous loops.

## Discussion

**Fig 11:**
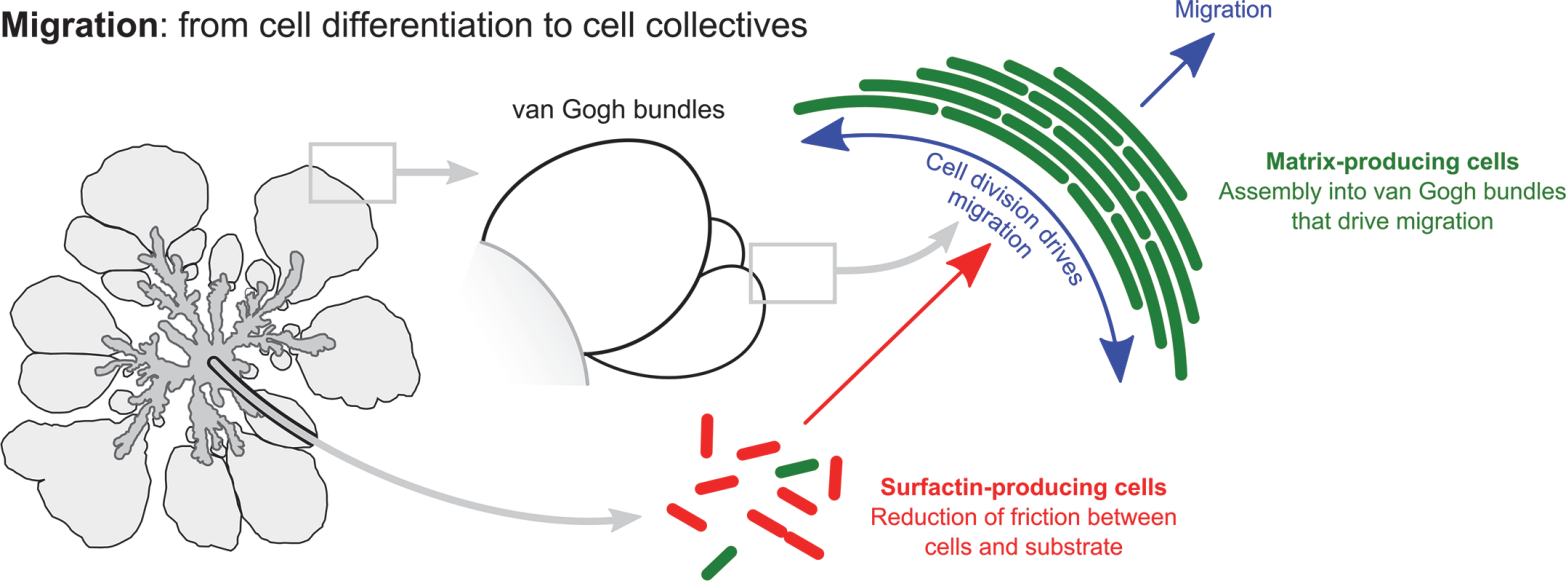
Schematic overview of cell differentiation and collective properties in *B*. *subtilis* colony expansion. Red and green cells represent, respectively, surfactin- and matrix-producing cells. Dendrites predominantly consist of surfactin-producing cells interspersed with clumps of matrix-producing cells. The petals of the colony consist predominantly of matrix-producing cells that form van Gogh bundles. We propose that surfactin mediates the expansion of van Gogh bundles by reducing the friction between the van Gogh bundles and substrate and that van Gogh bundle expansion is driven by cell division. The elastic and folding properties—dependent on matrix-producing cells—of the van Gogh bundles allow for an efficient colony expansion and prevent the bundles from breaking under increased compression.

In this study we analyzed sliding motility in *B*. *subtilis* to determine the factors that allow for the collective migration of cells. We found that cells organize themselves into bundles that spread by forming expanding filamentous loops at the colony edge. These cell collectives, which we call van Gogh bundles, are distinct from previously described filaments in *B*. *subtilis* due to their strong alignment and functionality [21,67]. The folding properties of the filamentous loops determine the migration rate and, in part, depend on the products secreted by matrix-producing cells. The development and expansion of van Gogh bundles depend critically on the synergic interaction of surfactin-producing and matrix-producing cells. To our knowledge, this is the first example of bacterial cells dividing labor in order to overcome one of the major ecological challenges: migration (Fig 11).

We show that colony expansion is characterized by up-regulation of *srfA* expression (i.e., the surfactin-producing cell type) followed by an increase in *tapA* expression (i.e., the matrix-producing cell type). The two expression phases correspond to the two growth periods that are apparent at the macroscopic level: dendrite formation and petal-shaped colony outgrowth [57]. The temporal dynamics in gene expression correspond to the regulatory pathways controlling cell differentiation in *B*. *subtilis*. For example, *srfA* expression is regulated by quorum sensing [41,68]; at high cell density, the expression of *srfA* increases, which explains the gradual up-regulation of *srfA* at the onset of colony growth. In addition, surfactin can function as a signal that triggers matrix production [41,42]. It is therefore not surprising that the peak in *srfA* expression is followed by a peak in *tapA* expression. This regulatory link between cell differentiation of surfactin-producing cells and matrix-producing cells corresponds closely to the functional link we describe in this study: van Gogh bundles, consisting of matrix-producing cells, can develop only in the presence of surfactin (Fig 11). Thus, surfactin-producing and matrix-producing cells divide labor in order to facilitate colony expansion (see also [69]). The division of labor typically evolves in response to strong phenotypic trade-offs [70,71]. For example, cyanobacteria divide labor between photosynthetic cells and heterocysts, because photosynthesis and nitrogen fixation are incompatible [72,73]. Likewise, there might be a trade-off between the formation of van Gogh bundles by matrix-producing cells and the production of surfactin. Unfortunately, it is unclear what this trade-off might be; perhaps the cell-to-cell attachment of matrix-producing cells would be harmed if cells simultaneously produced surfactin. The fact that *eps tasA* + *srfA* chimeras—colonies in which different strains perform different tasks—can expand further than WT colonies suggests there may indeed be a trade-off at play.

Besides surfactin, matrix production can also be triggered by environmental stressors like starvation, hypoxia, and osmotic stress [36,74,75]. Environmental changes during colony growth might therefore also be responsible for the temporal up-regulation of matrix production and the transition from the dendrite to the petal growth phase. We showed that cells isolated from the petal growth phase readily switch back to dendrite formation, when re-inoculated on a fresh growth medium. This indicates that the environment is indeed an important determinant for the different growth phases. When van Gogh bundles first appear, the matrix-producing cells are surrounded by surfactin-producing cells. Given their proximity, the co-occurring cell types probably sense nearly identical environmental conditions, yet they behave differently [69,76,77]. This indicates that—besides depending on the environment—cell differentiation also depends on inherent stochasticity. A recent study showed that under constant environmental conditions, cells can spontaneously differentiate into matrix-producing cell chains [78] that are preserved for a number of generations due to a regulatory feedback loop that creates a bi-stable switch [79–81]. A similar switch might also be important for the first cell chains that appear in the formation of van Gogh bundles.

While previous studies have shown that surfactin production and EPS production can affect colony expansion [47,48,58,59], these studies did not show a synergistic interaction between cell types. In addition, the colony expansion in our study is of a different nature than the ones described in previous studies. For example, EPS production has been shown to have a relatively small effect on biofilm colony expansion, and that effect was hypothesized to depend on osmotic pressures [47,48]. Here we show that EPS has an all-or-none effect on colony expansion during sliding motility. The migration of van Gogh bundles does not directly rely on osmotic gradients, but instead results from mechanic force (although osmotic gradients can affect cell differentiation [74]). Hence, EPS stimulates migration by allowing for the organization of van Gogh bundles. How EPS exactly guides bundle formation requires further examination. Our results suggest that EPS is required for side-to-side attachment of cell chains. However, EPS might also affect the pole-to-pole interactions. Besides being essential in the formation of van Gogh bundles, EPS production was also essential for dendrite formation. At this early growth phase, matrix-producing cells do form multicellular clumps, but these clumps lack the tight alignment of cells that characterizes the van Gogh bundles. Thus, the mere presence of surfactin-producing and matrix-producing cells does not guarantee the formation of van Gogh bundles. It would be interesting to examine why matrix-producing cells are essential for dendrite formation, while forming van Gogh bundles only in the petal growth phase.

The functions of EPS and TasA inside the van Gogh bundle are different. Whereas EPS is absolutely necessary for the formation of van Gogh bundles, TasA seems to fine-tune the folding properties of the van Gogh bundles. TasA specifically localizes to the pole-to-pole interaction zones of TasA-producing cells inside the van Gogh bundle. Our mathematical model shows that the folding properties of van Gogh bundles determine the efficiency of migration: when the filament is less likely to fold, it can expand farther in space. We suggest that TasA might affect folding, by manipulating the bending rigidity at the pole-to-pole interactions between cells. Although this claim awaits further biophysical quantification, our study suggests that both EPS and TasA have specialized functions that guide the development of van Gogh bundles [64–66]. In this way, matrix-producing cells can organize themselves into multicellular structures that facilitate migration.

*B*. *subtilis* is not the only species that switches to a multicellular lifestyle to accomplish migration. Filamentous structures also occur during the colony growth of *P*. *vortex* and *B*. *mycoides*, whose growth patterns are described in the Introduction [28–30]. Furthermore, an impressive study by Vilain and colleagues [34] showed that the closely related species *B*. *cereus* switches to a multicellular lifestyle when grown on filter-sterilized soil-extracted soluble organic matter (SESOM) or artificial soil microcosm (ASM)—media that mimic the environmental conditions cells encounter in the soil. They showed that the lifestyle switch to multicellularity allows for migration. Interestingly, *B*. *mycoides* and *B*. *subtilis* show the same lifestyle switch when exposed to SESOM or ASM. This strongly supports our hypothesis that the collective properties that emerge from the interaction between surfactin-producing and matrix-producing cells—van Gogh bundles—evolved to facilitate migration. This hypothesis is further supported by the fact that the domesticated lab strain, *B*. *subtilis* 168, which is known to be defective in surfactin production, cannot make the switch to a multicellular lifestyle when grown on SESOM or ASM [34,82]. It would be interesting to examine whether SESOM and ASM indeed induce surfactin and matrix production and hence the development of van Gogh bundles in the wild isolate of *B*. *subtilis*.

Like other forms of bacterial multicellularity [9], van Gogh bundles illustrate how the organization of cells can help to overcome important ecological challenges. Ultimately, we hope that the study of such simple forms of organization can improve our understanding on how evolution constructs [10,83–88]: how cells can evolve to become integrated collectives that, together, form a new organizational unit.

## Materials and Methods

### Experiments

#### Strains and medium

The complete strain list is shown in S1 Table. All strains were derived from the WT NCIB3610, which is a non-domesticated isolate [21]. Colonies were grown on a modified MSgg medium. MSgg medium is typically used to study *B*. *subtilis* biofilm or pellicle formation [21]. Fall and colleagues [57] adjusted the medium to study *B*. *subtilis* sliding. In contrast to swimming and swarming motility, sliding is a flagellum-independent type of motility that depends on cell division [89–92]. In this medium, MSggN, amino acids present in the MSgg medium were eliminated and KH_2_PO_4_ was replaced by NaH_2_PO_4_. In addition, it had been shown that *B*. *subtilis* sliding motility depends on the potassium concentration [59]; therefore, Fall and colleagues [57] examined two main variants of MSggN medium: one with a low and one with a high potassium concentration by adding either 100 μM or 5 mM KCl. In our study, we used the variant of MSggN with 100 μM KCl. Twenty milliliters of MSggN medium was used per petri dish (diameter = 9 cm); the medium was solidified by adding 0.6% agarose. Plates were prepared 1 d before inoculation and were poured at a fixed temperature of 65°C. Plates were turned upside down about 15–30 min after pouring them and left in a single layer at room temperature for 16–18 h before inoculating the cell culture. Plates were in a single layer when incubated in the 37°C room for the sliding assay. For each experiment fresh medium was prepared, using the same medium for all growth and replicate conditions.

#### Inoculation conditions

Two standard inoculation conditions were used for the experiments: plates were inoculated from overnight (O/N) colonies grown on MSggN (37°C) by either (1) toothpick inoculation or (2) pipet inoculation with 2 μl of colony suspension. For the colony suspension, the O/N colony was re-suspended in 300 μl of PBS, after which the cell density was normalized to an optical density (λ = 600 nm) of 2. In cases where cells were toothpick inoculated, this is mentioned in the figure caption. Since for these inoculation conditions cells came from O/N-grown colonies, some cells had already produced surfactin or matrix at the onset of colony growth. As a control, in which no surfactin- and matrix-producing cells were present in the inocula (e.g., used for the experiment associated with Fig 4), we applied a passaging method before inoculating cells onto the agar plate [93].

For passaging, cells were grown in 5-ml LB broth cultures for eight consecutive cycles using 16-ml test tubes (37°C). In the first cycle, cells were grown for 2 h, while for all other cycles, for 1.5 h. The first passaging cycle was initiated by cells from an O/N colony grown on MSggN (37°C). Then, the cell culture of the first growth cycle was used to inoculate the second, etcetera. At the onset of each new cycle, the cells were diluted to a target OD_600_ of 0.004. On average, cells divided 4.12 ± 0.36 (mean ± standard deviation) times per cycle, making the average doubling time 22 min. The optical density at the end of a cycle was on average 0.072 ± 0.017. Cell cultures were on average diluted 17.97 ± 4.32 times from one cycle to the next. Although similar passaging experiments had been shown to eliminate matrix-producing cells from the inocula [93], it was yet unclear whether the same would hold for surfactin-producing cells. Therefore, we monitored *tapA* and *srfA* expression (using fluorescent reporters in a double-labeled P_*tapA*_-CFP P_*srfA*_-YFP strain) for the different inoculation conditions (S15 Fig): the default inoculum condition, as described above, and different stages in the passaging experiment. Cell cultures at the end of cycle 6 (blue in S15 Fig) and 8 (purple) of the passaging experiment were examined, as well as two cases in which we did not transfer the cultures from one cycle to the next, but instead let them grow for three consecutive cycles without passaging (yellow and green in S15 Fig). As expected, the cell culture at the end of the passaging experiment (purple) did not have cells that produced surfactin or matrix, while the normal inoculum from O/N colonies (red) did. Passaging was essential to prevent cell differentiation, since cultures that grew for three consecutive cycles without re-inoculation showed strong cell differentiation (S15 Fig).

#### The preparation of colony samples for microscopy

For the time-course experiments shown in Fig 3, colonies were dispersed before data acquisition by microscopy. Colony samples were scraped from the agar plate and re-suspended in PBS; depending on the colony size, 100–1,000 μl of PBS was used. After this, the colony suspension was mixed using a vortex and, if there were still clumps left, treated by additional re-suspension with a syringe (23 gauge needle). To check fluorescence intensity profiles, 10 μl of the colony suspension was inoculated on an agarose patch that was solidified on a microscope slide (200 μl of 1.5% agarose in PBS flattened on a microscopy slide).

In cases where intact colony edges were examined by microscopy, a piece of the colony edge was cut from the agar plate. This piece was subsequently flipped onto a glass-bottom well (PELCO Glass Bottom Dish) and immediately analyzed under the microscope. In all cases, replicate samples were used from the same colony as well as from different colonies, to see how variable the observed cellular structures were inside the colony and between different colonies.

#### Microscopy, imaging, and image analysis

For imaging, four different devices were used: a digital camera with a macro lens, a stereomicroscope, an upright microscope, and an inverted microscope (see below for product details). All high-magnification imaging at the cellular level was done with the inverted microscope (60× magnification). High-magnification images were used for gene expression analyses. The fluorescence intensities in microscope images were analyzed using a standard procedure: (1) images were taken using constant fluorescent exposure times, (2) images were loaded and segmented in a MatLab program [62,94], and (3) pixel data were written to text files that could be analyzed with available statistics software (in this study, R; http://www.r-project.org/). All images were included in the analysis, except for a few images that were out of focus. For details on the image analysis software we used see [62,94].

#### Product specifications

For plate imaging, a Nikon D7000 camera was used with an AF Micro Nikkor 60-mm macro lens. The stereomicroscope was a Zeiss Stemi SV6 stereomicroscope equipped with a 1.0× Achromat S objective lens and an AxioCam charge-coupled device (CCD) video camera system (Zeiss). For imaging, we used Axiovision suite software from Zeiss. The upright microscope was a Zeiss Axioskop 2 Plus equipped with an A-Plan 10× objective and a long-distance Plan-NEOFLUAR 20× objective. The camera was an AxioCam MRc (Zeiss) with Axiovision software to capture images. The inverted microscope was a Nikon Eclipse TE2000-U microscope equipped with a 20× Plan Apo objective and a 60× Plan Apo oil objective. Pictures were made with a Hamamatsu digital camera, model ORCA-ER. Filter sets were from Chroma, model #52017 (CFP-YFP dual-band filter), and model #62002v2 (DAPI/FITC/Texas Red).

### Model

We constructed a model to study how cellular properties could influence features of multicellular organization such as the folding properties of the van Gogh bundles, which are important for the rate of colony expansion. We did not aim to accurately model the biophysical details of the growth of van Gogh bundles (parameterization of such a model would be impossible), but rather to make a simple phenomenological model to shape our intuition based on previous models of epithelium folding [65,66]. In the model we examine the growth of a cellular filament. Unlike the van Gogh bundles, the filament is simplified to a single chain of pole-to-pole-attached cells. The cells in the filament can elongate, divide, and turn and thereby affect the macroscopic shape of the filament. The filament is placed in a two-dimensional space with fixed boundaries (the space is 1 × 1 spatial units big; this size is relevant for the cell size and growth rate mentioned below). The cells inside the filament are not allowed to overlap, and the ends of the filament are fixed in space, as being attached to the colony. At the start of each simulation, the filament consists of *N* cells that are placed as a horizontal line at the bottom of the two dimensional space (*y-*coordinate is 0). The filament is updated every time step by selecting a random cell from the population and performing one out of three possible update events: (1) cell elongation, (2) cell division, or (3) cell turning. After *T* time steps the simulation is stopped. The final shape of the filament results from the accumulation of local update events. The degree of colony expansion is measured in the *y*-direction. Here we give a short description for each of the three update events (see S16 Fig).

#### Event 1: Cell elongation

Cells grow with rate *R* per time step. *R* is taken from a uniform distribution ranging from 0 to *G* (i.e., the maximal growth rate). The average growth rate of a cell is therefore ½*G*. Since cells occur inside a filament, cell elongation affects the spatial orientation of cells. We assume that a cell grows in the direction of one of its neighboring cells (this neighbor is randomly selected). When a cell elongates, it pushes its neighboring cell away, both cells thereby change their relative position. Cell elongation is illustrated in the upper panel of S16 Fig.

#### Event 2: Cell division

When a cell exceeds the maximum cell size, *S*, it divides. The mother cell is divided in two equally long daughter cells. Division results in a new filament junction. Importantly, during the process of cell division the spatial orientation of the daughter cells is identical to that of the mother cell (S16 Fig). In other words, the filament does not change its shape during cell division. The cell size is checked after each cell elongation event, thereby assuring that no cells are larger than the maximum cell size.

#### Event 3: Cell turning

Perhaps the most intricate update event is cell turning. During an event of cell turning, the focal cell changes its spatial orientation with respect to its neighboring cells. The spatial orientation of a cell is adjusted in two steps. First a potential new spatial orientation is generated by randomly turning one of a focal cell’s neighboring cells with an angle β (taken from a normal distribution with mean 0 and standard deviation *B*), thereby adjusting as well the orientation of the focal cell itself and of the focal cell’s other neighboring cell. Second, the potential new spatial orientation is compared with the old one, and the new spatial orientation is adopted only if it is energetically favorable.

The spatial configuration of cells is associated with a certain energy state, which is the so-called potential energy that is stored in the current state of the system (i.e., bending energy). Cells are expected to change their spatial configuration such that the potential energy is minimized. The potential energy relates to the spatial orientation of cells in the following way (see S16 Fig for the angles):

$$V={\left(\text{π}-{\text{α}}_{1}\right)}^{2}+{\left(\text{π}-{\text{α}}_{2}\right)}^{2}$$ (1)

*V* is the potential energy, α_1_ and α_2_ are the angles a cell makes with its left and right neighbors, and π is the angle between neighboring cells (i.e., 180°, no angle) that would minimize the potential energy that is stored in the system. The potential energy of the current (*V*_c_) and new (*V*_n_) spatial orientation are compared to calculate the chance of switching. When *V*_n_ < *V*_c_, the probability that the cells turn from the current to the new spatial orientation is given by

$$P=1-e^{k\left(V_{\text{c}}-V_{\text{n}}\right)}$$ (2)

*P* is the chance that a cell turns to its new spatial orientation, and *k* is a parameter that determines the bending rigidity. When *k* is high, a drop in the potential energy (*V*_n_ < *V*_c_) is more likely to result in a re-orientation of the cells, thereby reducing the angle between a cell and its neighbors. In other words, cells are considered resistant against bending (i.e., high bending rigidity) when they are likely to reduce the angle between them and their neighbors (i.e., minimizing the local curvature).

In the main text we consider three different parameter settings (S2 Table): (1) default setting, (2) high bending rigidity, and (3) large cell size. It is currently impossible to quantitatively compare our experimental results with the simulation outcomes, because an accurate parameterization of the model is not possible. It is however possible to qualitatively compare the different parameter settings with the experimental results obtained by microscopy. See S2 Table for all the parameter values that were used for the presented simulations.

## Supporting Information

S1 Data Data of figures. This is an excel file containing the data of Figs 3, 10, S2, S9–S11, S13, and S15.
(XLSX)

S1 Fig Toothpick re-inoculation of cells from morphologically distinct parts of the colony. Cells transferred from the morphologically distinct regions of a WT colony to fresh medium: samples 1 and 2 are taken from dendrites, samples 3 and 4 are taken from petals, and samples 5 and 6 are taken from rays. Top: locations of colony from which samples were collected. Bottom: the colonies produced by the re-inoculated colony samples after 1 d of growth on a fresh medium. Despite some small differences in colony size, all re-inoculated colonies are morphologically the same. Toothpick inoculation was chosen to minimize manipulation of samples during re-inoculation.
(TIFF)

S2 Fig Co-expression of *srfA* and *tapA* in a wild-type strain. Gene expression of *srfA* and *tapA* is monitored by YFP and CFP fluorescence intensities, respectively (P_*srfA*_-YFP and P_*tapA*_-CFP). For each combination of fluorescence intensities, the ratio between the observed and expected pixel frequency is shown (see S1 Text). Fluorescence intensity combinations to which more pixels belong than expected by chance are colored dark blue, while those to which fewer pixels belong than expected by chance are colored cyan (those with the expected number of pixels are colored white). When no pixels are observed, the fluorescence intensity combination is colored grey. The graph does not show the density of pixels over the different fluorescence intensities. The microscopy pictures used for this analysis were also used for the time-course experiment in Fig 3.
(TIFF)

S3 Fig Measurement of cell alignment. Here we show the two steps that underlie the quantification of cell alignment. In the first step, cells are segmented using advanced image analysis software, MicrobeTracker [94], thereby determining a cell’s outline and major axis. The major axis is divided in approximately equally sized cell segments to account for the curvature of a cell. In the second step, the alignment of cell segments is determined by comparing the spatial orientation of the focal cell segment with that of its neighbors (excluding segments that belong to the same cell as the focal cell segment). The neighborhood includes all segments that are within a radius of 20 pixels of the focal cell segment.
(TIFF)

S4 Fig Cell orientation in a population of single cells and van Gogh bundles. Top: phase-contrast images of cells at the colony edge in the dendrite growth phase (left, single cells) and the petal growth phase (right, van Gogh bundles). Bottom: superimposed coloration that shows the spatial orientation of cells. The color shows the angle of cell segments (see S2 Text for details). Regions from the microscopy image in which cells could not be accurately tracked (e.g., overlapping cells), were excluded from the analysis.
(TIFF)

S5 Fig Level of alignment in a population of single cells and van Gogh bundles. This figure shows the level of alignment between cells for the microscopy images shown in S4 Fig A low level of alignment indicates that cells are oriented in different directions (blue) and a high level of alignment indicates that cells are oriented in the same direction (white); see S2 Text for details on alignment measurement. Top: alignment at the colony edge in the dendrite growth phase (left, single cells) and the petal growth phase (right, van Gogh bundles). Bottom: vector fields, showing the spatial orientation of cells, superimposed on alignment plots.
(TIFF)

S6 Fig Cell orientation and alignment in a mixed population of single cells and van Gogh bundles. Analysis of the phase-contrast image of Fig 7A shows a mixed population of single cells and van Gogh bundles (*n* = 1,930 cells). Top: phase-contrast image. Top right: spatial orientation of cells (for legend see S4 Fig). Bottom left: level of alignment in the population (for legend see S5 Fig). Bottom right: vector field superimposed on the alignment plot, showing clear distinction between regions with and without van Gogh bundles. Regions in the microscopy image in which cells could not be accurately tracked (e.g., overlapping cells and parts of cells at the image edge) were excluded from the analyses.
(TIFF)

S7 Fig Distribution of angular differences between a focal cell segment and neighboring cell segments. The dark and light blue lines (*n* = 5,590 cells) and dark and light red lines (*n* = 2,751 cells) show the average distribution of angular differences between neighboring cell segments for populations of single cells and van Gogh bundles, respectively (see S2 Text for details on calculation). Each distribution is based on all the angular differences between the focal cell segments and their neighbors within an image (using 10% of all cell segments). The distributions are plotted in bins of 9°, so the first bin includes angular differences of 0–9° between neighboring cell segments, the second bin includes angular differences of 9–18°, etc. The plot inset shows the average shape of a cell that is part of a van Gogh bundle or a population of single cells (based on phase-contrast images), accounting for the average cell length, cell curvature, and cell alignment with respect to neighboring cells. The average angle between neighboring cells inside van Gogh bundles and in a population of single cells is 4.5° and 21°, respectively.
(TIFF)

S8 Fig Chimeric colonies in transition between dendrite and petal growth phase. Here are the colonies of four mutant chimeras a few hours before the microscopy images shown in Fig 6 were taken: (1) *srfA* + *eps*, (2) *srfA* + *tasA*, (3) *eps* + *tasA*, (4) *eps tasA* + *srfA*. Images in Fig 6 are taken at the colony edge. As shown in Fig 2A, colony expansion is slightly slower in *srfA + tasA* and *eps + tasA* mutant chimeras than in *srfA + eps* and *eps tasA + srfA* mutant chimeras.
(TIFF)

S9 Fig TasA concentration at the boundary between van Gogh bundles and surrounding single cells. Left: phase-contrast and fluorescence images of Fig 7A. The image section that is scrutinized in detail is included in the rectangle. Top right: magnification of the section in the phase-contrast image that is subject to detailed analysis, showing van Gogh bundle on the left side and single cells on the right side. Middle right: average angle between neighboring cell segments across the image section. Cells on the left side, corresponding to the van Gogh bundle, are strongly aligned (i.e., small angular differences), and cells on the right side are weakly aligned (i.e., large angular differences). Bottom right: TasA fluorescence across image section. The red dots show the fluorescence intensity of the pixels, the thick black line shows the average intensity along the image cross-section and the thin black lines show the standard deviation. Peaks in fluorescence intensities correspond to pole-to-pole interactions between cells. Fluorescence values are normalized towards background fluorescence.
(TIFF)

S10 Fig TasA distribution at pole-to-pole and side-to-side cell interactions. Left: phase-contrast and fluorescence images of van Gogh bundles of the TasA-mCherry strain (similar to those shown in Fig 7A). Superimposed on the phase-contrast image are the line segments along which TasA fluorescence is determined. The major axis line segments correspond to line segments along a cell’s major axis at the cell poles (pole-to-pole interactions). The minor axis line segments correspond to line segments along a cell’s minor axis at the cell sides (side-to-side interactions). Each line segment functions as a transect along which the TasA fluorescence intensity is measured. Right: fluorescence intensities along line segments. The transparent red lines show the fluorescence intensities along each major axis line segment (*n* = 311), and the transparent blue lines show the fluorescence intensities along each minor axis line segment (*n* = 363). The bold thick and thin lines show the average fluorescence intensity and standard deviation, respectively. Since the line segments differ in length, they are centralized around the highest fluorescence value that is measured along the line segment, which is set to pixel location 0. The symmetry of the fluorescence distributions shows that the highest fluorescence values are in the middle of the line segments—i.e., the intercellular space between cells.
(TIFF)

S11 Fig TasA fluorescence at pole-to-pole interactions of wild-type and *tasA* mutant cells in a van Gogh bundle. Left: phase-contrast and fluorescence images of a chimeric van Gogh bundle consisting of WT TasA-mCherry cells and mutant *tasA*-YFP cells (similar to the chimera shown in Fig 7B). The fluorescence image is a composite image showing mutant cells (artificially colored green) and localization of TasA protein (red fluorescence). The phase-contrast image shows the van Gogh bundle. Superimposed are line segments corresponding to the pole-to-pole interactions between WT cells (red, *n* = 460) and between mutant cells (blue, *n* = 192). Along these line segments the TasA fluorescence intensity is determined, as was done for the major and minor axis line segments in S10 Fig Right: fluorescence intensities along line segments. Transparent lines correspond to the fluorescence intensities along the individual line segments. The bold thick and thin lines correspond to the average fluorescence intensity and standard deviation, respectively.
(TIFF)

S12 Fig Colony expansion of wild-type and *srfA* colonies when exogenous surfactin is added. Colony growth of WT (upper images) and *srfA* mutant (lower images) without adding solution prior to inoculation (left), with adding 10 μl of 20 mM NaOH solution prior to inoculation (middle), and with adding 10 μl of surfactin solution (10 mg/ml surfactin in 20 mM NaOH solution) (right) prior to inoculation [58]. All plates were inoculated with colony suspensions with standardized cell density (see Materials and Methods and [58]).
(TIFF)

S13 Fig Folding properties of filamentous loops in wild-type and *tasA* mutant colonies. The folding properties of the outermost loops at the colony edge of WT and *tasA* colonies are characterized by the distribution of angles. A segmented line is drawn on top of each loop, with regularly sized line segments (accomplished by a mesh overlay). The angles between the neighboring line segments determine the folding properties of a filamentous loop. *tasA* loops have more and stronger folds than WT loops, as is apparent from the distributions of angles; the relative angles between line segments in *tasA* loops are smaller (Mann Whitney *U* test: *p* < 10^−16^, *W* = 206,266). Five microscopy images were examined for each strain, resulting in 509 and 625 concatenated line segments in *tasA* mutant and WT loops, respectively.
(TIFF)

S14 Fig Artificial induction of *tasA* expression and colony expansion. *tasA* transcription was artificially induced in an IPTG-inducible *tasA* strain by adding 0, 0.05, 0.1, and 0.2 mM IPTG to growth medium (MSggN). Romero and colleagues [50] showed that the WT biofilm morphology on MSgg (similar to our growth medium, MSggN) can be recovered in an IPTG-inducible *tasA* strain by adding 0.2 mM IPTG. Colony morphology is not recovered by adding IPTG to our growth medium (i.e., dendrites are lacking), but colony expansion is recovered (i.e., not considering morphology) when 0.05 mM IPTG or more is added.
(TIFF)

S15 Fig Effect of passaging on the composition of cell types in the initial inoculum. (A) CFP and YFP fluorescence intensities correspond respectively to *tapA* and *srfA* expression in a P_*tapA*_-CFP P_*srfA*_-YFP WT strain. The range of fluorescence intensities was measured for six different cell cultures: (1) non-labeled WT (control) (black), (2) cell culture form O/N colony grown on MSggN (37°C) (red), (3) cell culture at the end of cycle 6 during passaging (blue), (4) cell culture at the end of cycle 8 during passaging (purple), (5) cell culture that grew for three consecutive cycles without passaging starting in cycle 4 (yellow), and (6) cell culture that grew for three consecutive cycles without passaging starting in cycle 6 (green). (B) Schematic representation of passaging experiment, including some exemplary microscopy pictures that were used for the fluorescence analysis (only phase-contrast pictures are shown).
(TIFF)

S16 Fig Schematic overview of events that can occur during filament growth in the model. Three cellular events can occur: (1) cell elongation, (2) cell division, and (3) cell turning. Cells are shown as rectangles, with red lines through their major axes and red dots at the pole ends. The red circles surrounding the cells help to determine the spatial orientation of cells. For example, during elongation a cell becomes longer, but also the spatial orientation of cells changes in accordance with the new intersection point between the corresponding red circles (compare dashed and solid red circles in the upper right panel). The spatial orientation of cells during cell division remains unaltered. Cell turning depends on the angle between the focal cell and its neighbors (α_1_ and α_2_). Cells turn if the randomly generated new orientation is energetically favorable compared to the original orientation (see model description in Materials and Methods for details).
(TIFF)

S17 Fig Phase-contrast microscopy images used for analysis in S7 Fig Top: phase-contrast microscopy images of van Gogh bundles at the colony edge. Bottom: phase-contrast microscopy images of a population of single cells at the colony edge, early during colony development.
(TIFF)

S1 Movie Colony expansion and van Gogh bundles in the second growth period. The colony was imaged using an upright microscope for a period of 3 h (10-min intervals) at the colony edge. The filamentous loops consist of van Gogh bundles.
(MOV)

S1 Table Strain list. (DOCX)

S2 Table Parameter settings of the model. (DOCX)

S1 Text Characterization of cell types: co-expression of *srfA* and *tapA*. (DOCX)

S2 Text Quantification of van Gogh bundles. (DOCX)

S3 Text TasA distribution. (DOCX)

## Abbreviations

ASM: artificial soil microcosm
EPS: extracellular polysaccharide
O/N: overnight
PBS: phosphate buffered saline
SESOM: soil-extracted soluble organic matter
WT: wild type
