# Supplementary figures and images for: From Cell Differentiation to Cell Collectives: Bacillus subtilis Uses Division of Labor to Migrate
Source: PLoS Biol. 2015 Apr 20;13(4):e1002141. doi: 10.1371/journal.pbio.1002141 (PMC4403855; doi:10.1371/journal.pbio.1002141)

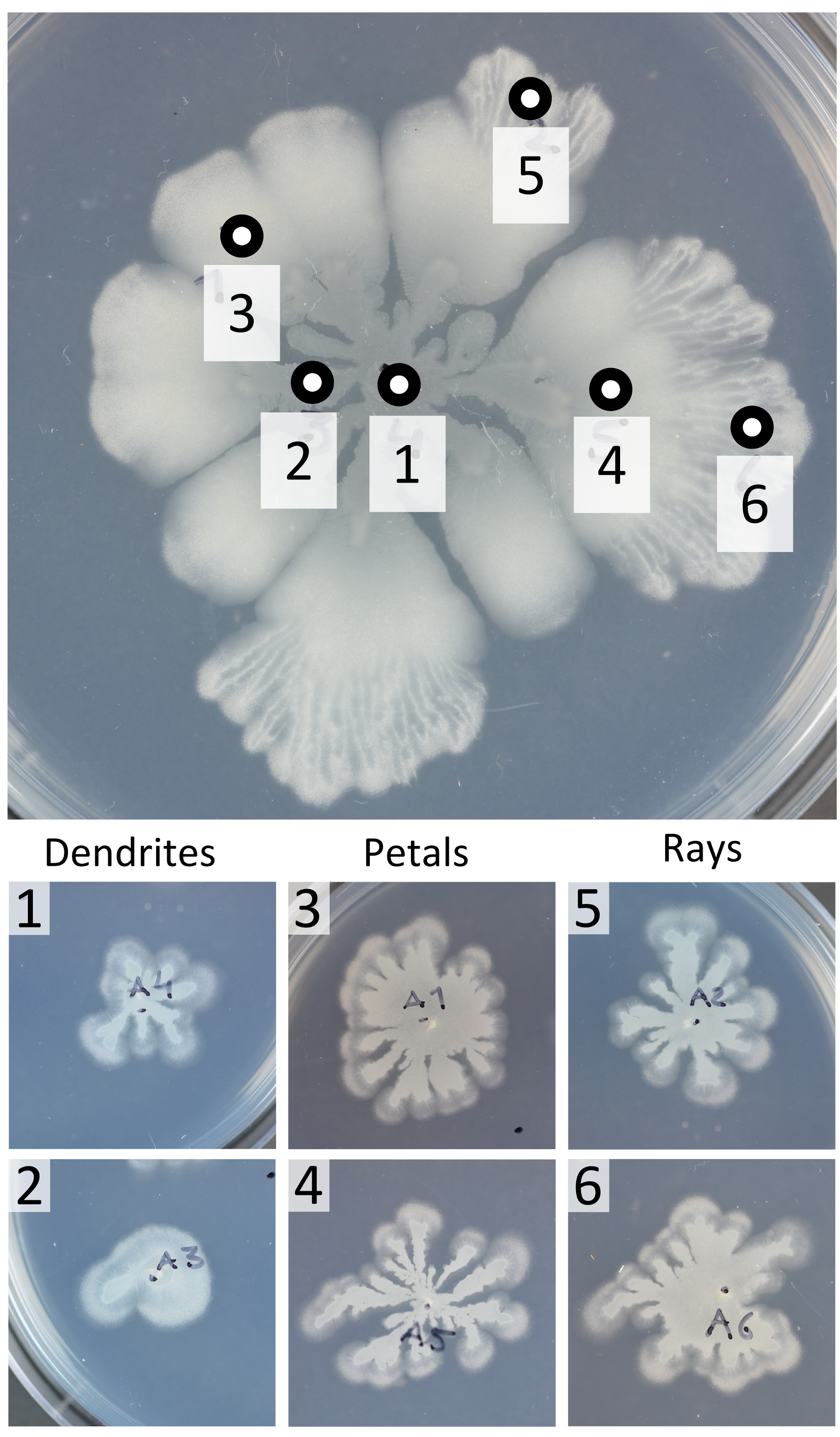

Supplement: S1 Fig — Cells transferred from the morphologically distinct regions of a WT colony to fresh medium: samples 1 and 2 are taken from dendrites, samples 3 and 4 are taken from petals, and samples 5 and 6 are taken from rays. Top: locations of colony from which samples were collected. Bottom: the colonies produced by the re-inoculated colony samples after 1 d of growth on a fresh medium. Despite some small differences in colony size, all re-inoculated colonies are morphologically the same. Toothpick inoculation was chosen to minimize manipulation of samples during re-inoculation. (TIFF) [file pbio.1002141.s002.tiff]

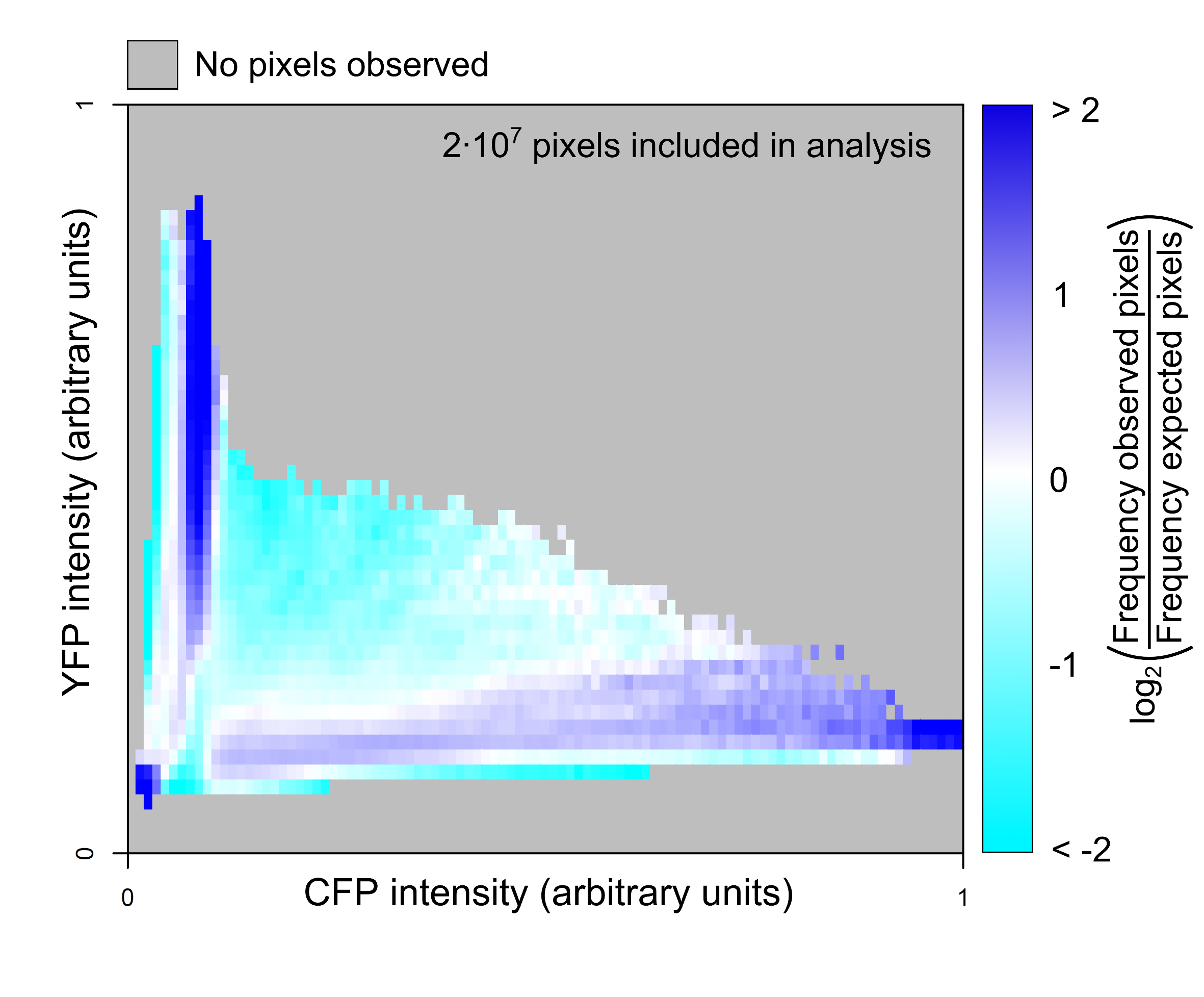

Supplement: S2 Fig — Gene expression of srfA and tapA is monitored by YFP and CFP fluorescence intensities, respectively (PsrfA-YFP and PtapA-CFP). For each combination of fluorescence intensities, the ratio between the observed and expected pixel frequency is shown (see S1 Text). Fluorescence intensity combinations to which more pixels belong than expected by chance are colored dark blue, while those to which fewer pixels belong than expected by chance are colored cyan (those with the expected number of pixels are colored white). When no pixels are observed, the fluorescence intensity combination is colored grey. The graph does not show the density of pixels over the different fluorescence intensities. The microscopy pictures used for this analysis were also used for the time-course experiment in Fig 3. (TIFF) [file pbio.1002141.s003.tiff]

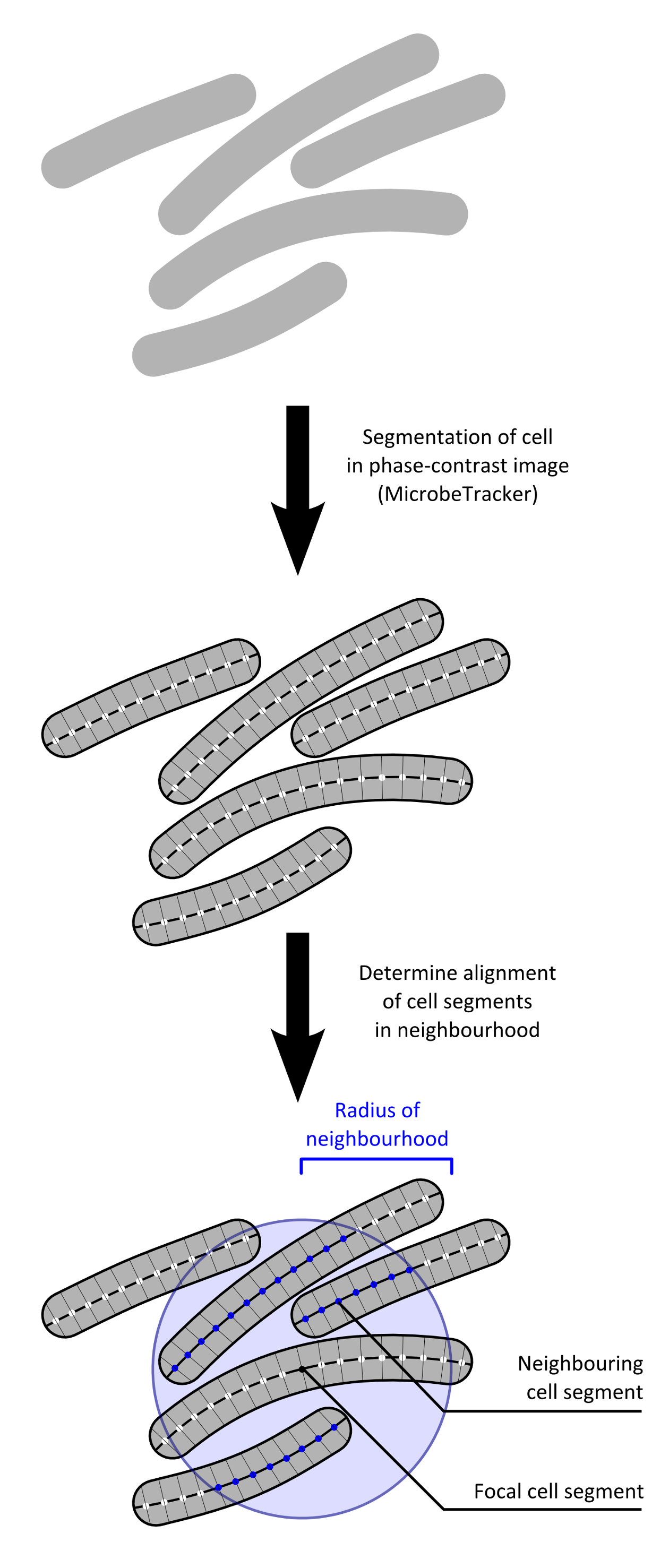

Supplement: S3 Fig — Here we show the two steps that underlie the quantification of cell alignment. In the first step, cells are segmented using advanced image analysis software, MicrobeTracker [94], thereby determining a cell’s outline and major axis. The major axis is divided in approximately equally sized cell segments to account for the curvature of a cell. In the second step, the alignment of cell segments is determined by comparing the spatial orientation of the focal cell segment with that of its neighbors (excluding segments that belong to the same cell as the focal cell segment). The neighborhood includes all segments that are within a radius of 20 pixels of the focal cell segment. (TIFF) [file pbio.1002141.s004.tiff]

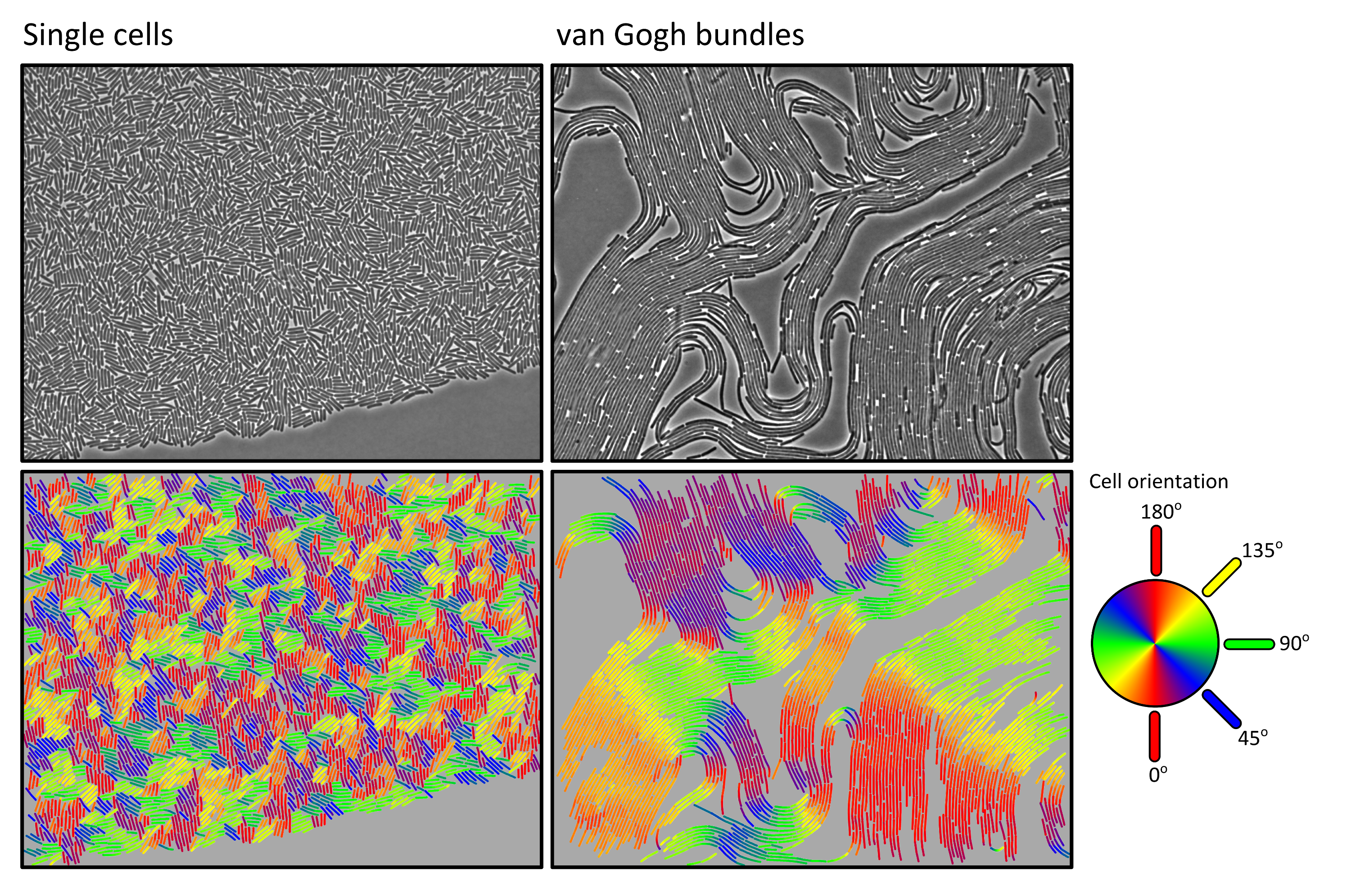

Supplement: S4 Fig — Top: phase-contrast images of cells at the colony edge in the dendrite growth phase (left, single cells) and the petal growth phase (right, van Gogh bundles). Bottom: superimposed coloration that shows the spatial orientation of cells. The color shows the angle of cell segments (see S2 Text for details). Regions from the microscopy image in which cells could not be accurately tracked (e.g., overlapping cells), were excluded from the analysis. (TIFF) [file pbio.1002141.s005.tiff]

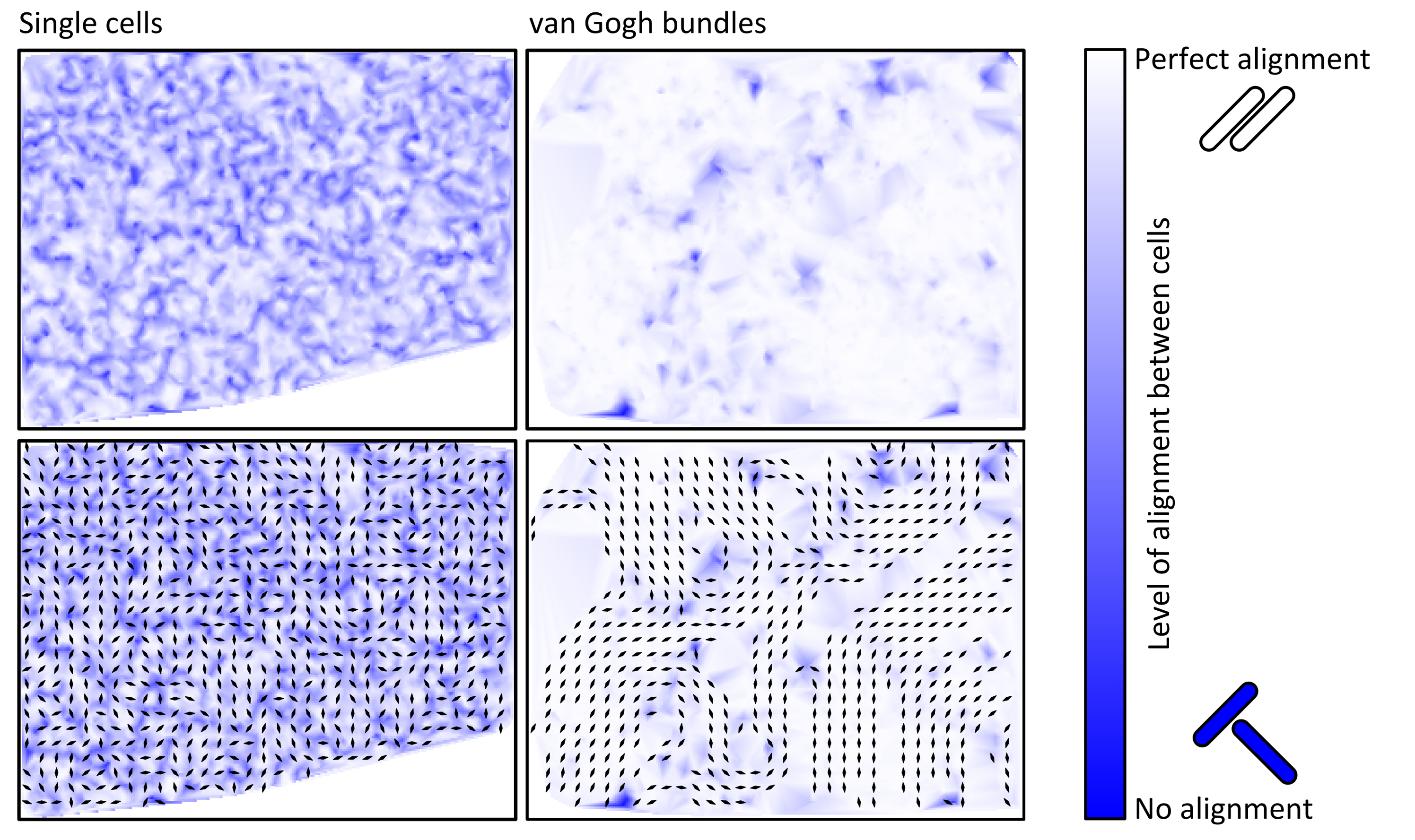

Supplement: S5 Fig — This figure shows the level of alignment between cells for the microscopy images shown in S4 Fig A low level of alignment indicates that cells are oriented in different directions (blue) and a high level of alignment indicates that cells are oriented in the same direction (white); see S2 Text for details on alignment measurement. Top: alignment at the colony edge in the dendrite growth phase (left, single cells) and the petal growth phase (right, van Gogh bundles). Bottom: vector fields, showing the spatial orientation of cells, superimposed on alignment plots. (TIFF) [file pbio.1002141.s006.tiff]

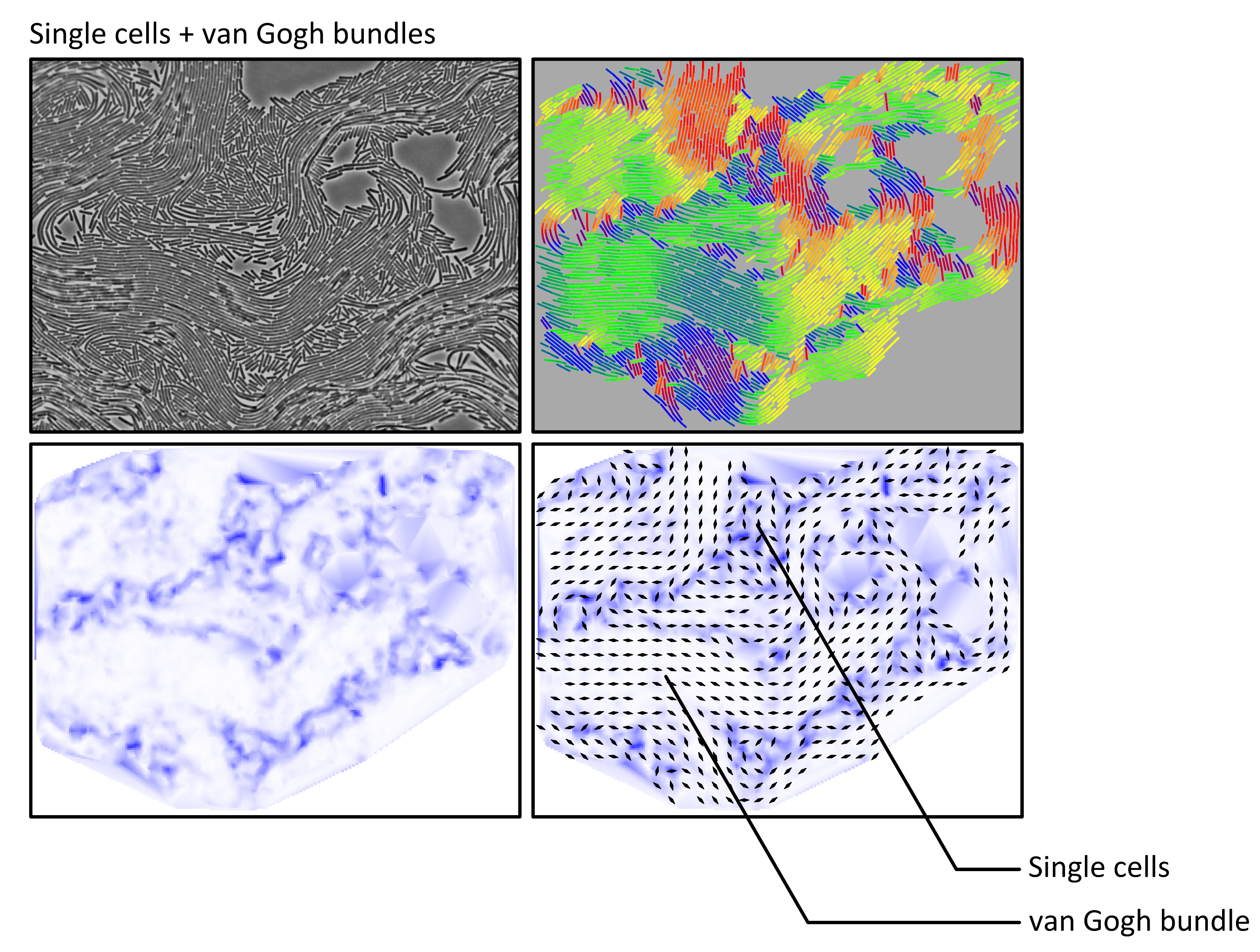

Supplement: S6 Fig — Analysis of the phase-contrast image of Fig 7A shows a mixed population of single cells and van Gogh bundles (n = 1,930 cells). Top: phase-contrast image. Top right: spatial orientation of cells (for legend see S4 Fig). Bottom left: level of alignment in the population (for legend see S5 Fig). Bottom right: vector field superimposed on the alignment plot, showing clear distinction between regions with and without van Gogh bundles. Regions in the microscopy image in which cells could not be accurately tracked (e.g., overlapping cells and parts of cells at the image edge) were excluded from the analyses. (TIFF) [file pbio.1002141.s007.tiff]

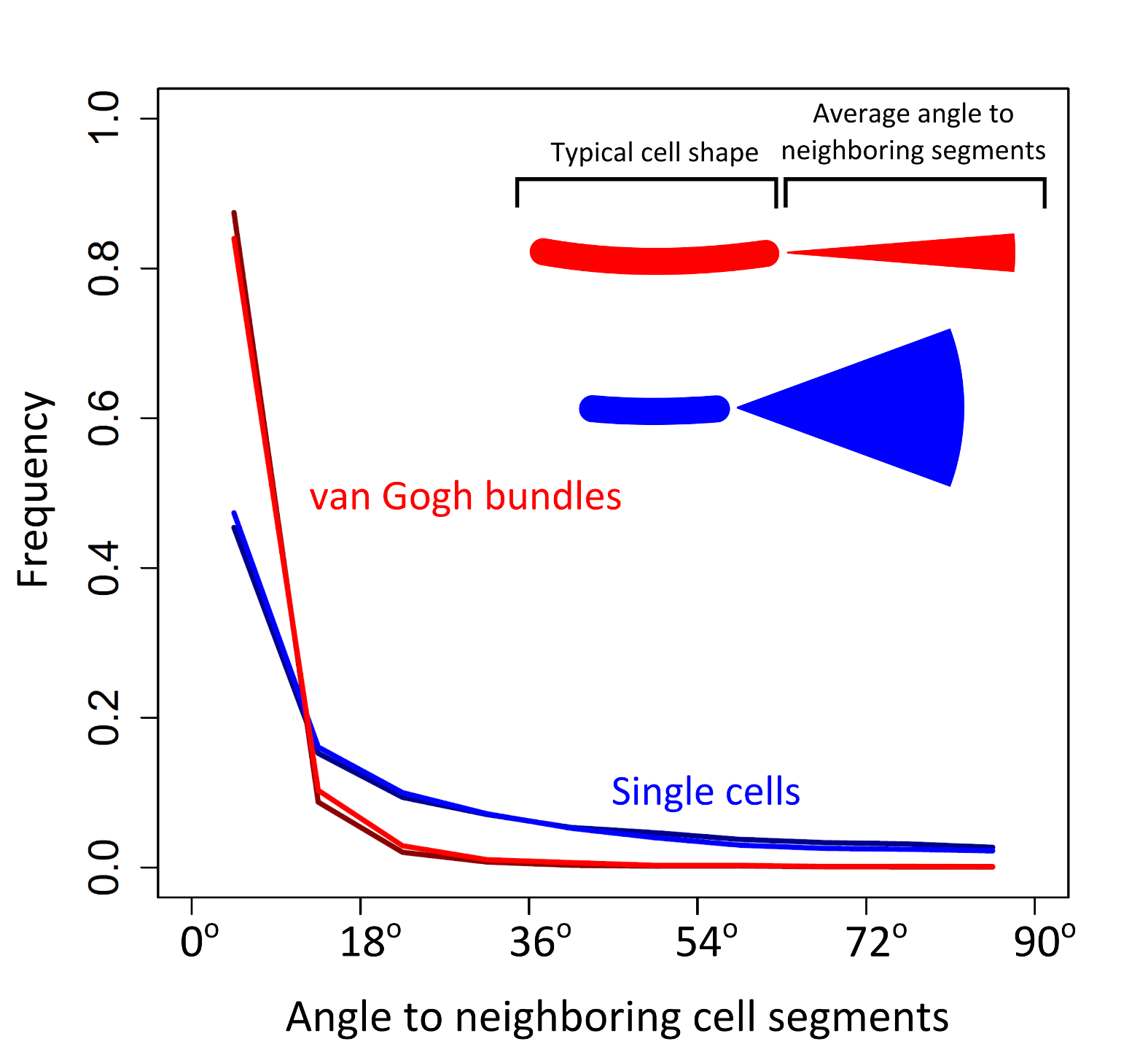

Supplement: S7 Fig — The dark and light blue lines (n = 5,590 cells) and dark and light red lines (n = 2,751 cells) show the average distribution of angular differences between neighboring cell segments for populations of single cells and van Gogh bundles, respectively (see S2 Text for details on calculation). Each distribution is based on all the angular differences between the focal cell segments and their neighbors within an image (using 10% of all cell segments). The distributions are plotted in bins of 9°, so the first bin includes angular differences of 0–9° between neighboring cell segments, the second bin includes angular differences of 9–18°, etc. The plot inset shows the average shape of a cell that is part of a van Gogh bundle or a population of single cells (based on phase-contrast images), accounting for the average cell length, cell curvature, and cell alignment with respect to neighboring cells. The average angle between neighboring cells inside van Gogh bundles and in a population of single cells is 4.5° and 21°, respectively. (TIFF) [file pbio.1002141.s008.tiff]

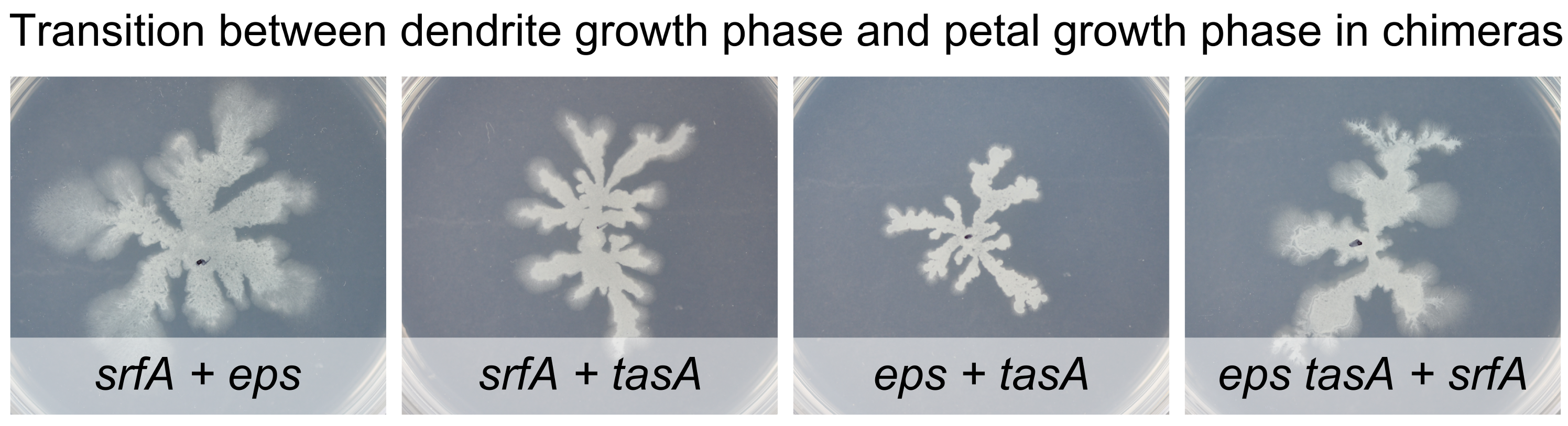

Supplement: S8 Fig — Here are the colonies of four mutant chimeras a few hours before the microscopy images shown in Fig 6 were taken: (1) srfA + eps, (2) srfA + tasA, (3) eps + tasA, (4) eps tasA + srfA. Images in Fig 6 are taken at the colony edge. As shown in Fig 2A, colony expansion is slightly slower in srfA + tasA and eps + tasA mutant chimeras than in srfA + eps and eps tasA + srfA mutant chimeras. (TIFF) [file pbio.1002141.s009.tiff]

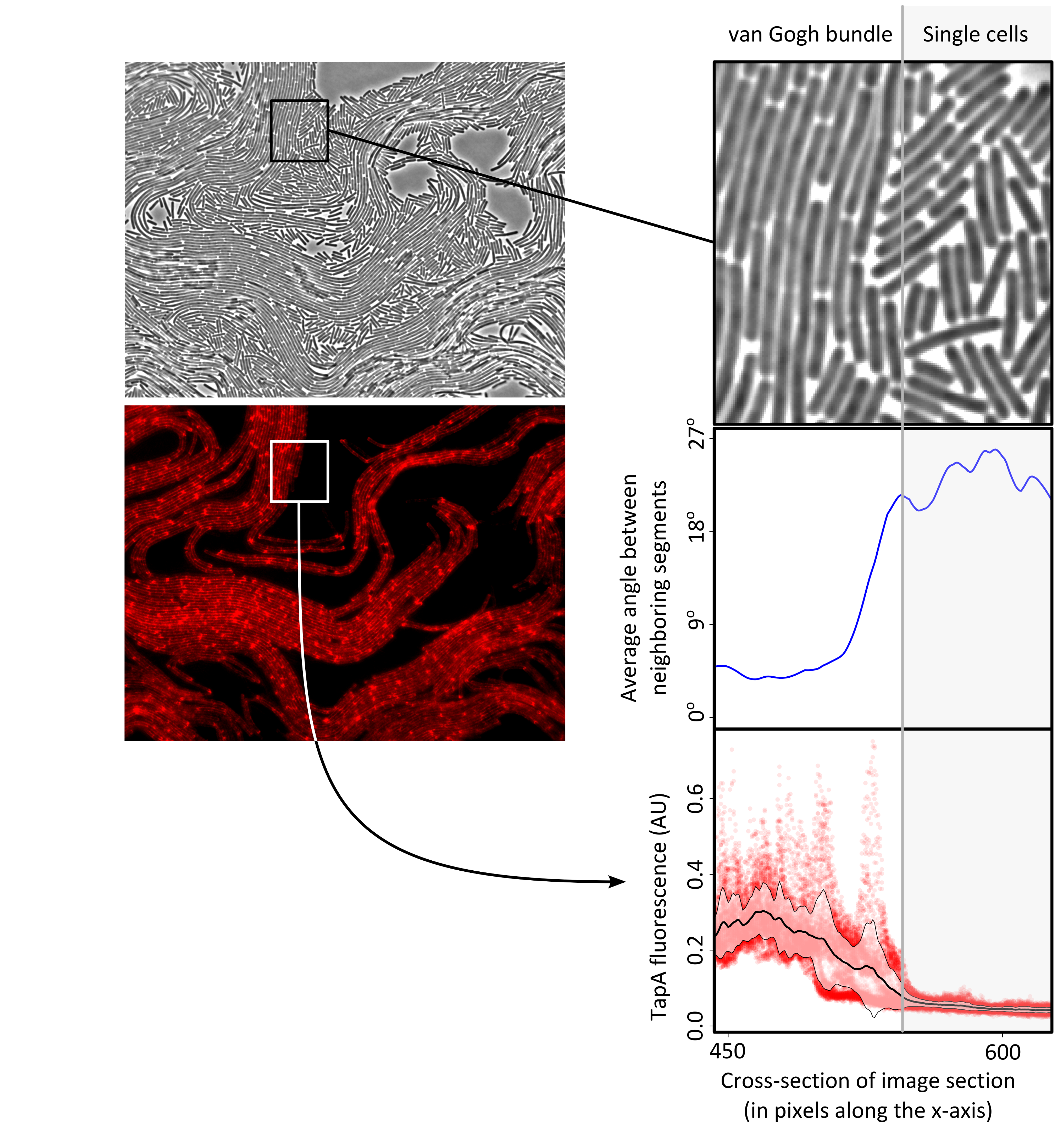

Supplement: S9 Fig — Left: phase-contrast and fluorescence images of Fig 7A. The image section that is scrutinized in detail is included in the rectangle. Top right: magnification of the section in the phase-contrast image that is subject to detailed analysis, showing van Gogh bundle on the left side and single cells on the right side. Middle right: average angle between neighboring cell segments across the image section. Cells on the left side, corresponding to the van Gogh bundle, are strongly aligned (i.e., small angular differences), and cells on the right side are weakly aligned (i.e., large angular differences). Bottom right: TasA fluorescence across image section. The red dots show the fluorescence intensity of the pixels, the thick black line shows the average intensity along the image cross-section and the thin black lines show the standard deviation. Peaks in fluorescence intensities correspond to pole-to-pole interactions between cells. Fluorescence values are normalized towards background fluorescence. (TIFF) [file pbio.1002141.s010.tiff]

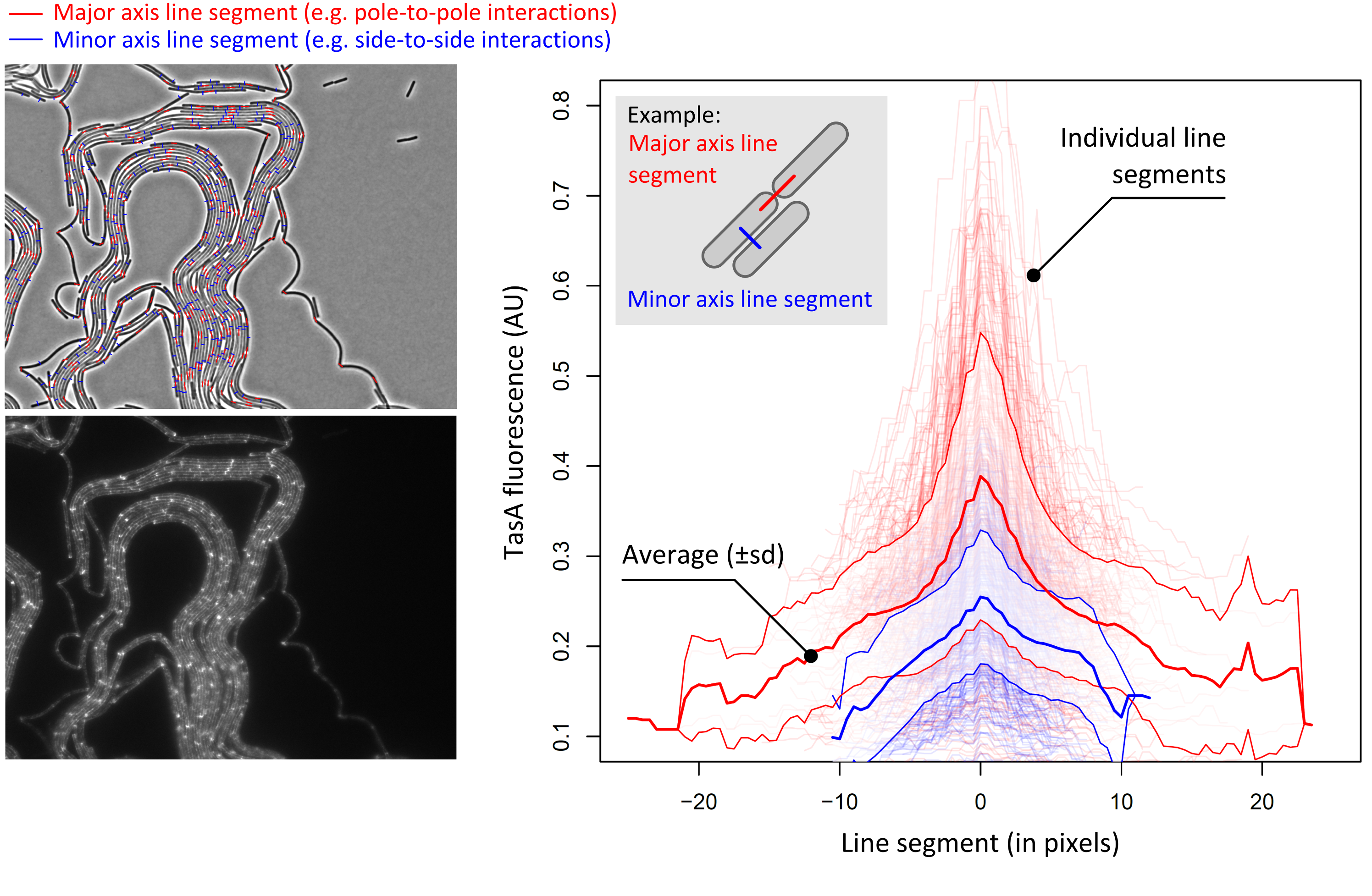

Supplement: S10 Fig — Left: phase-contrast and fluorescence images of van Gogh bundles of the TasA-mCherry strain (similar to those shown in Fig 7A). Superimposed on the phase-contrast image are the line segments along which TasA fluorescence is determined. The major axis line segments correspond to line segments along a cell’s major axis at the cell poles (pole-to-pole interactions). The minor axis line segments correspond to line segments along a cell’s minor axis at the cell sides (side-to-side interactions). Each line segment functions as a transect along which the TasA fluorescence intensity is measured. Right: fluorescence intensities along line segments. The transparent red lines show the fluorescence intensities along each major axis line segment (n = 311), and the transparent blue lines show the fluorescence intensities along each minor axis line segment (n = 363). The bold thick and thin lines show the average fluorescence intensity and standard deviation, respectively. Since the line segments differ in length, they are centralized around the highest fluorescence value that is measured along the line segment, which is set to pixel location 0. The symmetry of the fluorescence distributions shows that the highest fluorescence values are in the middle of the line segments—i.e., the intercellular space between cells. (TIFF) [file pbio.1002141.s011.tiff]

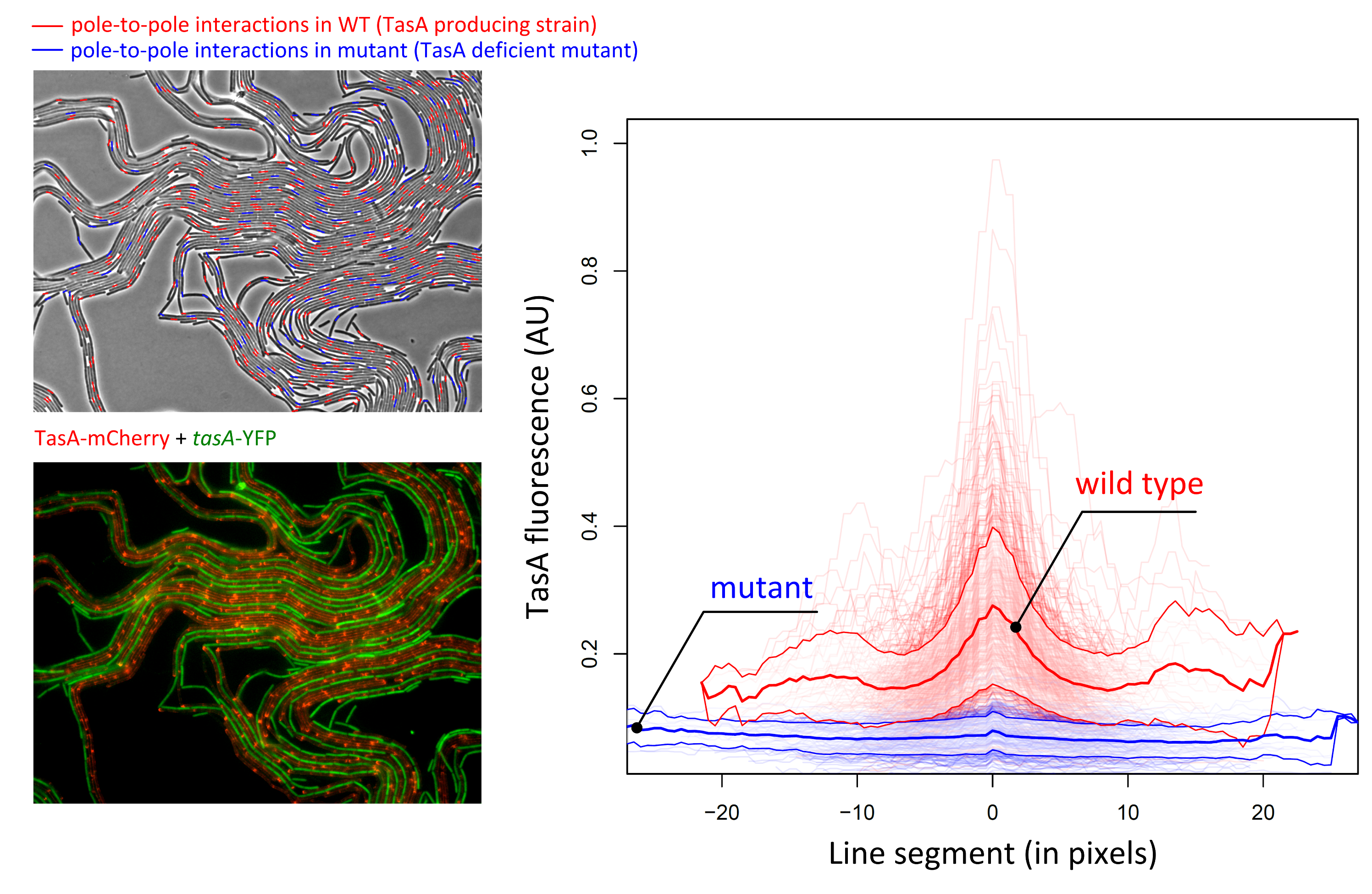

Supplement: S11 Fig — Left: phase-contrast and fluorescence images of a chimeric van Gogh bundle consisting of WT TasA-mCherry cells and mutant tasA-YFP cells (similar to the chimera shown in Fig 7B). The fluorescence image is a composite image showing mutant cells (artificially colored green) and localization of TasA protein (red fluorescence). The phase-contrast image shows the van Gogh bundle. Superimposed are line segments corresponding to the pole-to-pole interactions between WT cells (red, n = 460) and between mutant cells (blue, n = 192). Along these line segments the TasA fluorescence intensity is determined, as was done for the major and minor axis line segments in S10 Fig Right: fluorescence intensities along line segments. Transparent lines correspond to the fluorescence intensities along the individual line segments. The bold thick and thin lines correspond to the average fluorescence intensity and standard deviation, respectively. (TIFF) [file pbio.1002141.s012.tiff]

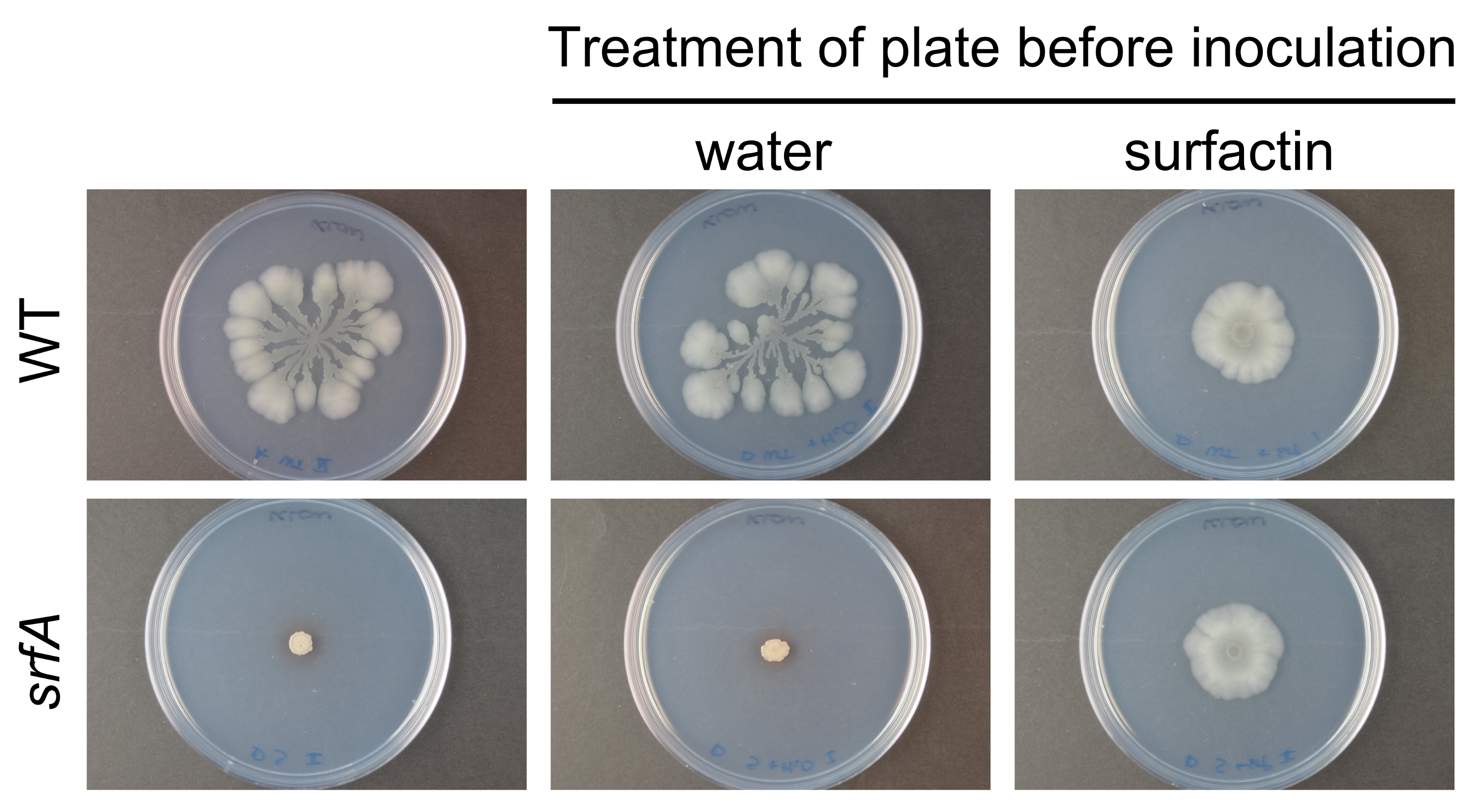

Supplement: S12 Fig — Colony growth of WT (upper images) and srfA mutant (lower images) without adding solution prior to inoculation (left), with adding 10 μl of 20 mM NaOH solution prior to inoculation (middle), and with adding 10 μl of surfactin solution (10 mg/ml surfactin in 20 mM NaOH solution) (right) prior to inoculation [58]. All plates were inoculated with colony suspensions with standardized cell density (see Materials and Methods and [58]). (TIFF) [file pbio.1002141.s013.tiff]

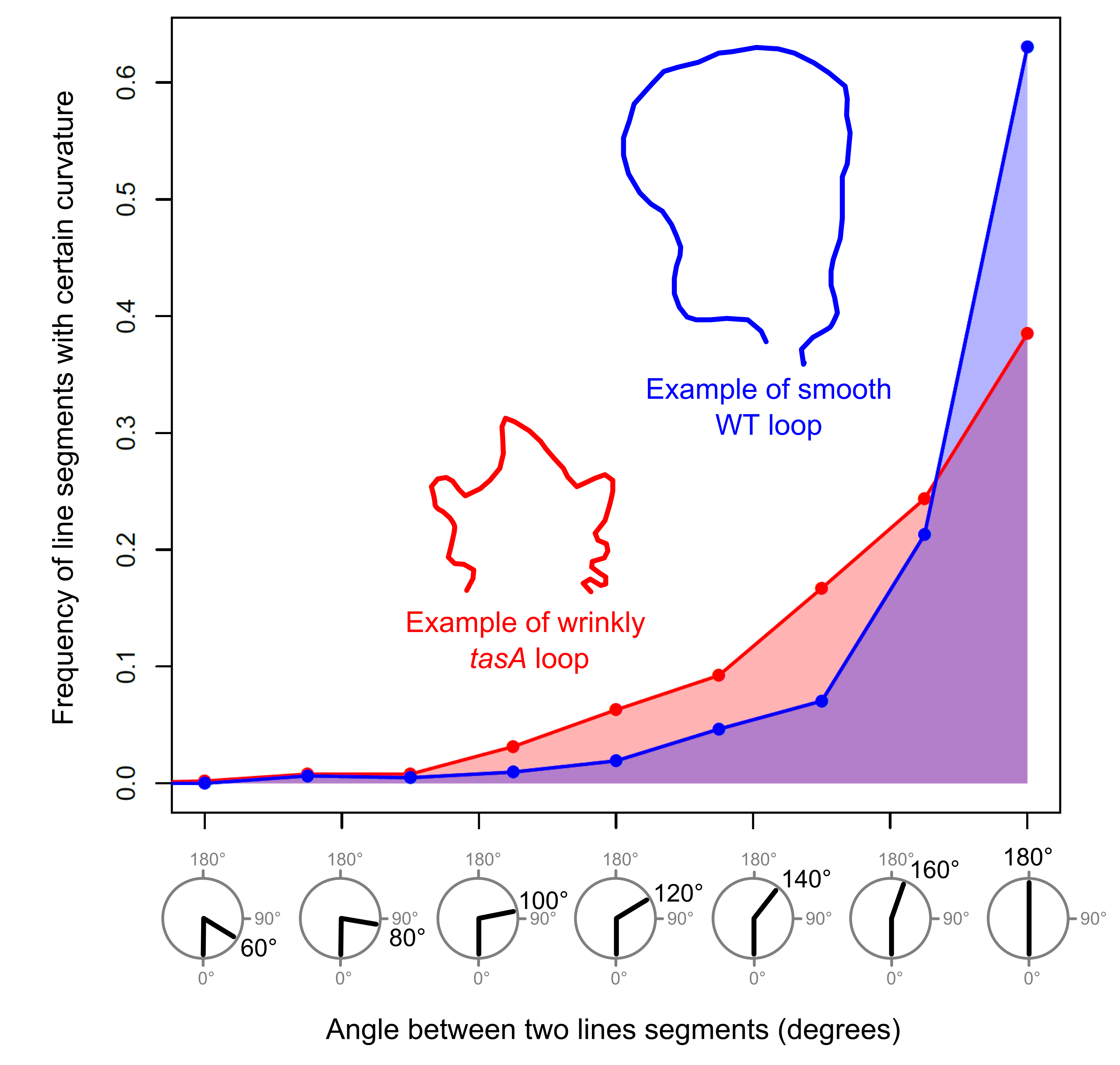

Supplement: S13 Fig — The folding properties of the outermost loops at the colony edge of WT and tasA colonies are characterized by the distribution of angles. A segmented line is drawn on top of each loop, with regularly sized line segments (accomplished by a mesh overlay). The angles between the neighboring line segments determine the folding properties of a filamentous loop. tasA loops have more and stronger folds than WT loops, as is apparent from the distributions of angles; the relative angles between line segments in tasA loops are smaller (Mann Whitney U test: p < 10−16, W = 206,266). Five microscopy images were examined for each strain, resulting in 509 and 625 concatenated line segments in tasA mutant and WT loops, respectively. (TIFF) [file pbio.1002141.s014.tiff]

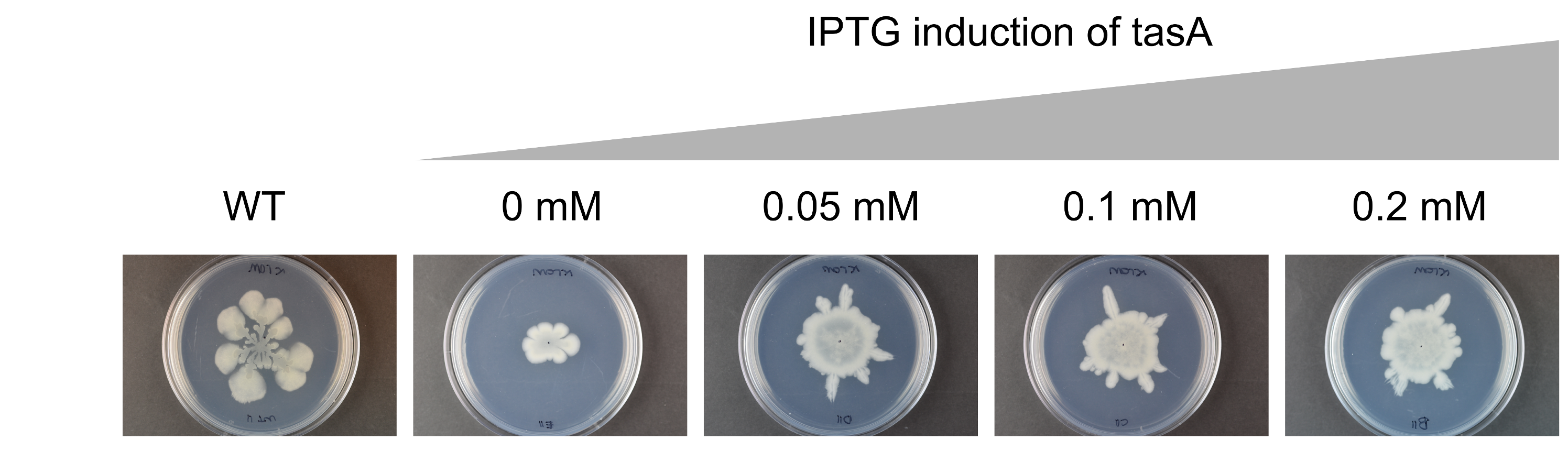

Supplement: S14 Fig — tasA transcription was artificially induced in an IPTG-inducible tasA strain by adding 0, 0.05, 0.1, and 0.2 mM IPTG to growth medium (MSggN). Romero and colleagues [50] showed that the WT biofilm morphology on MSgg (similar to our growth medium, MSggN) can be recovered in an IPTG-inducible tasA strain by adding 0.2 mM IPTG. Colony morphology is not recovered by adding IPTG to our growth medium (i.e., dendrites are lacking), but colony expansion is recovered (i.e., not considering morphology) when 0.05 mM IPTG or more is added. (TIFF) [file pbio.1002141.s015.tiff]

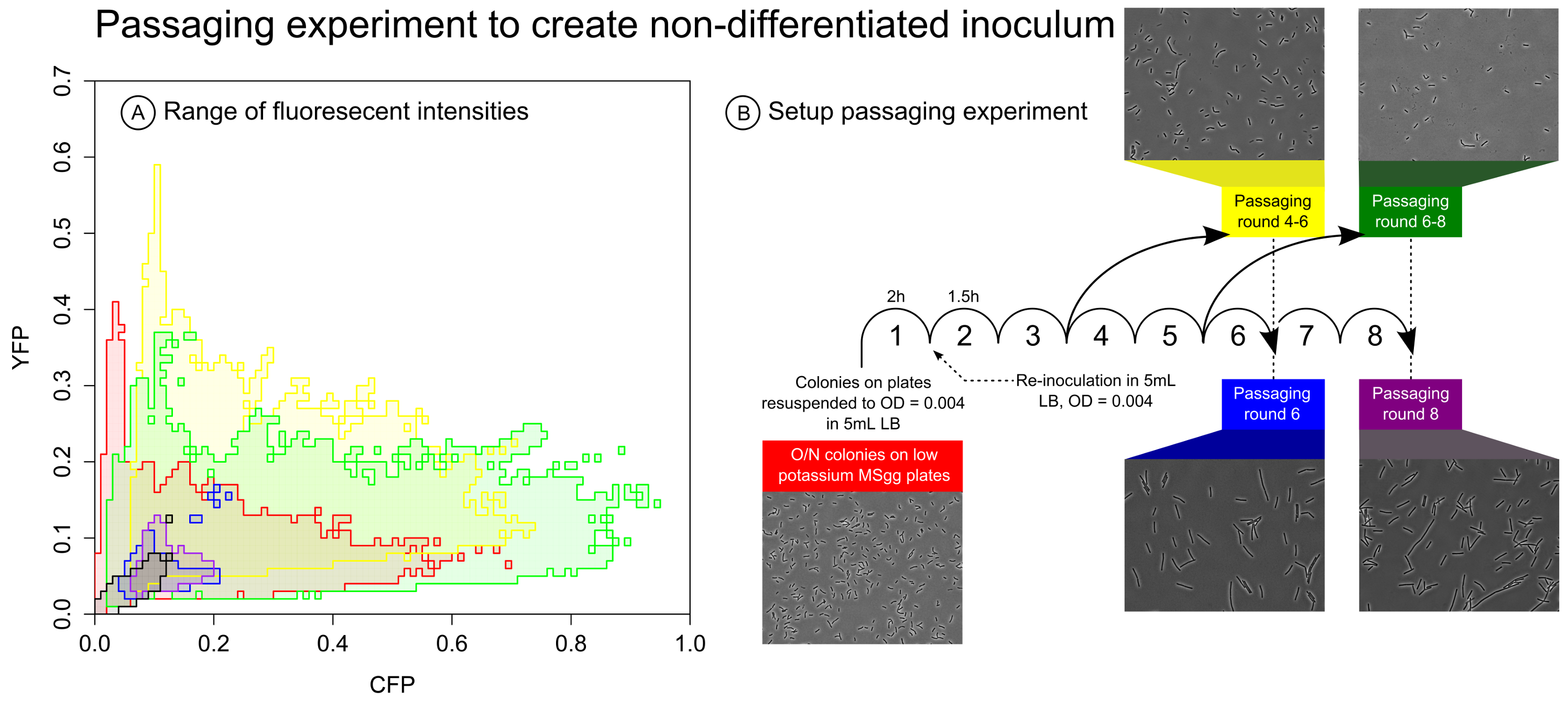

Supplement: S15 Fig — (A) CFP and YFP fluorescence intensities correspond respectively to tapA and srfA expression in a PtapA-CFP PsrfA-YFP WT strain. The range of fluorescence intensities was measured for six different cell cultures: (1) non-labeled WT (control) (black), (2) cell culture form O/N colony grown on MSggN (37°C) (red), (3) cell culture at the end of cycle 6 during passaging (blue), (4) cell culture at the end of cycle 8 during passaging (purple), (5) cell culture that grew for three consecutive cycles without passaging starting in cycle 4 (yellow), and (6) cell culture that grew for three consecutive cycles without passaging starting in cycle 6 (green). (B) Schematic representation of passaging experiment, including some exemplary microscopy pictures that were used for the fluorescence analysis (only phase-contrast pictures are shown). (TIFF) [file pbio.1002141.s016.tiff]

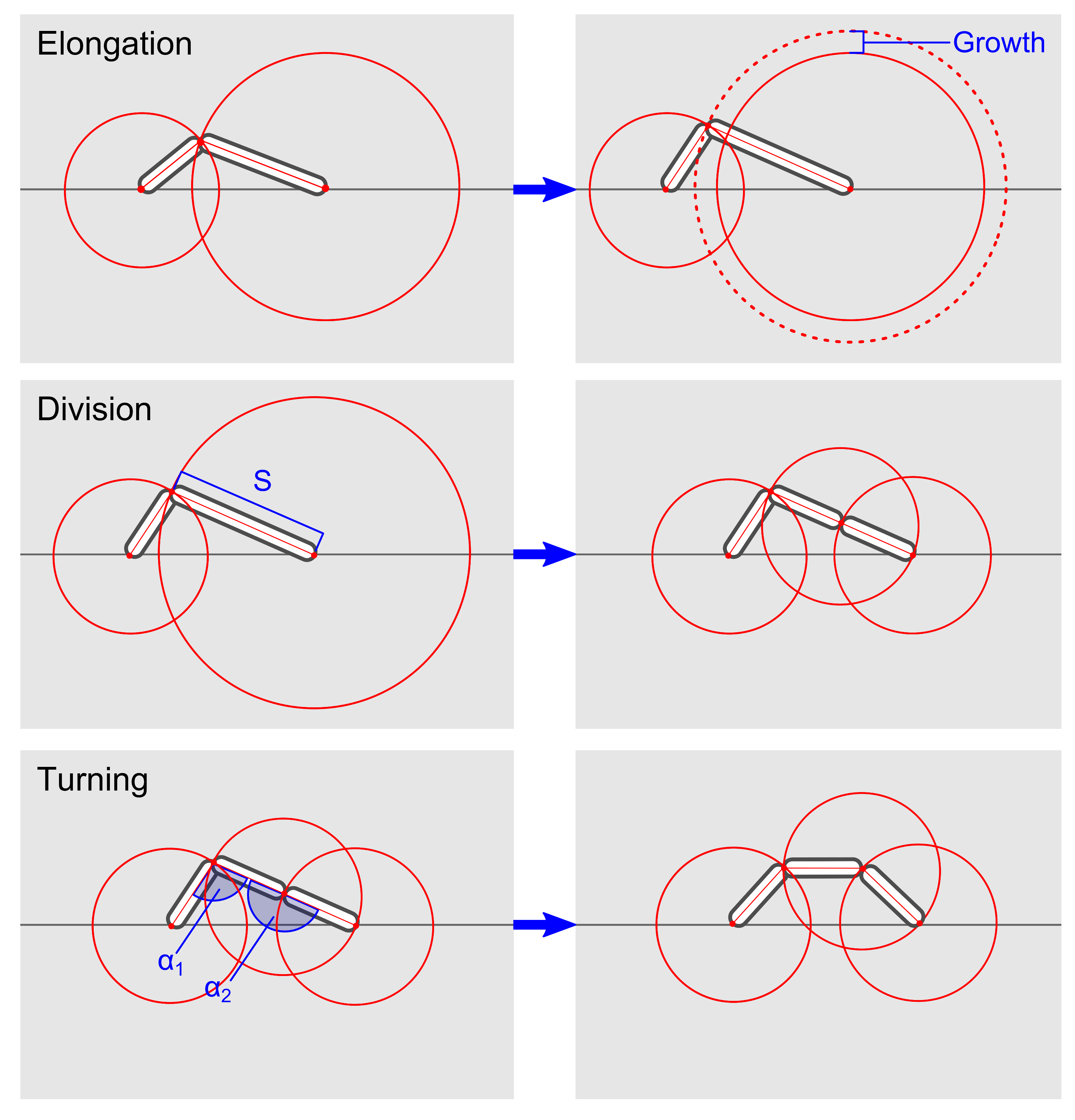

Supplement: S16 Fig — Three cellular events can occur: (1) cell elongation, (2) cell division, and (3) cell turning. Cells are shown as rectangles, with red lines through their major axes and red dots at the pole ends. The red circles surrounding the cells help to determine the spatial orientation of cells. For example, during elongation a cell becomes longer, but also the spatial orientation of cells changes in accordance with the new intersection point between the corresponding red circles (compare dashed and solid red circles in the upper right panel). The spatial orientation of cells during cell division remains unaltered. Cell turning depends on the angle between the focal cell and its neighbors (α1 and α2). Cells turn if the randomly generated new orientation is energetically favorable compared to the original orientation (see model description in Materials and Methods for details). (TIFF) [file pbio.1002141.s017.tiff]

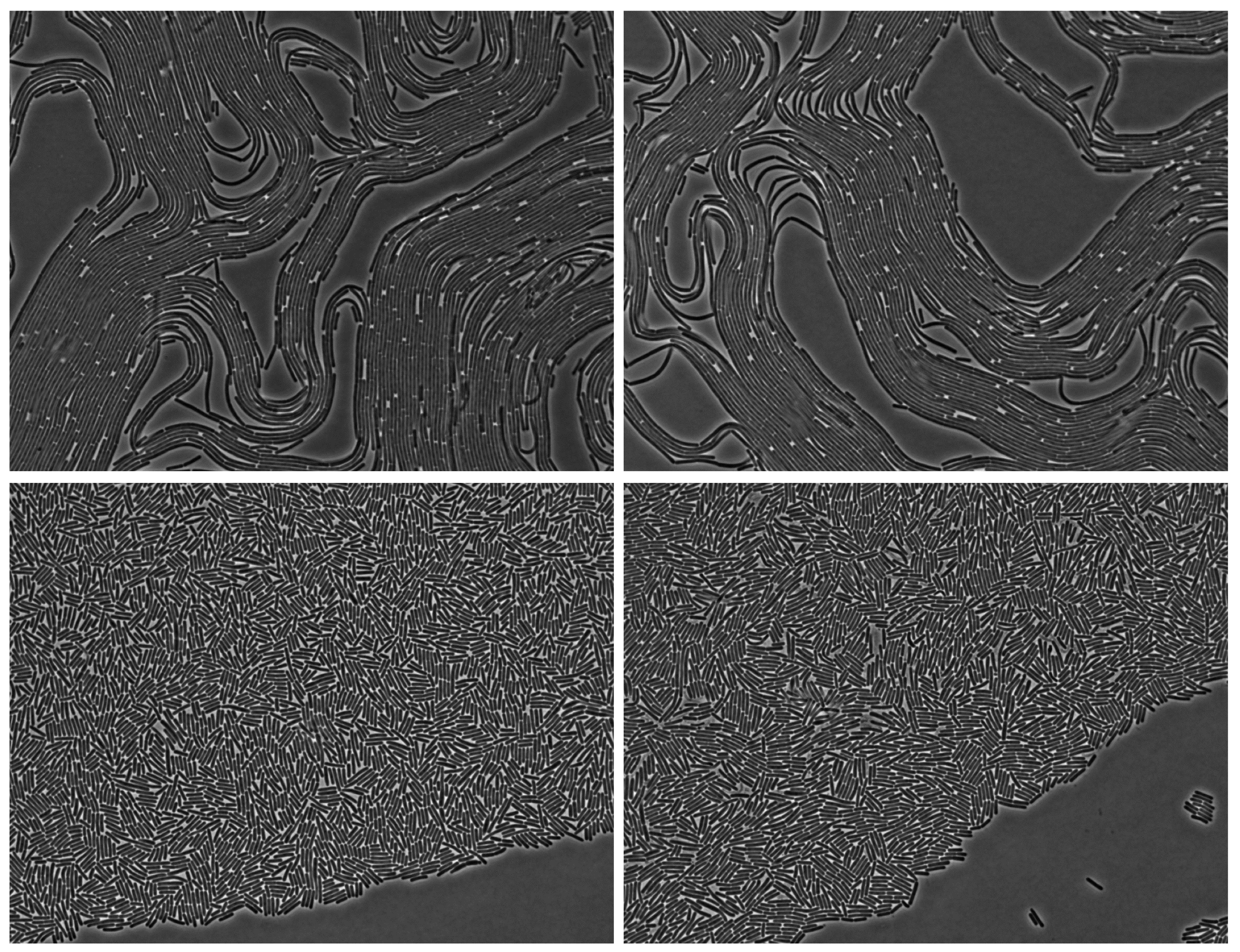

Supplement: S17 Fig — Bottom: phase-contrast microscopy images of a population of single cells at the colony edge, early during colony development. (TIFF) [file pbio.1002141.s018.tiff]
