# Supplementary material for: From Cell Differentiation to Cell Collectives: Bacillus subtilis Uses Division of Labor to Migrate
Source: PLoS Biol. 2015 Apr 20;13(4):e1002141. doi: 10.1371/journal.pbio.1002141 (PMC4403855; doi:10.1371/journal.pbio.1002141)
Supplement: S1 Table — (DOCX) [file pbio.1002141.s020.docx]

**Table S1.** Strain list

| **#** | **Name** | **Strain** | **Genotype*** | **Source** |
| --- | --- | --- | --- | --- |
| 1 | WT | NCIB 3610 | Undomesticated WT strain | Lab stock |
| 2 | *srfA* | ZK3858 | *srfAA*::erm | (1) |
| 3 | *tasA* | CA017 | *tasA*::kan | (2) |
| 4 | *eps* | SSB488 | *epsA-O*::tet | (3) |
| 5 | *eps tasA* | HV1235 | *epsA-O*::tet, *tas*::kan | (4) |
| 6 | *sigF* | CA002 | *sigF*::kan | (2) |
| 7 | *hag* | HV1150 | *hag*::tet | (2) |
| 8 | WT- mKate2 | NL020 | *amyE*::P*_hyperspank_*-mKate2 (cm) | NL |
| 9 | *srfA*-YFP | PB283 | *srfAA*::mls, *amyE*::P*_hyperc1o1_*-yfp (spec) | (5) |
| 10 | *srfA*- mKate2 | NL069 | *srfAA::*erm*, amyE::*P*_hyperspank_*-mKate2 *(cm)* | NL |
| 11 | *tasA*-YFP | PB229 | *tasA*::mls, *amy*::P*_hyperc1o1_*-yfp (spec) | (5) |
| 12 | *eps*-YFP | PB228 | *epsA-O*::tet, *amyE*::P*_hyperc1o1_*-yfp (spec) | (5) |
| 13 | *eps*- mKate2 | NL070 | *epsA-O::*tet*, amyE::*P*_hyperspank_*-mKate2 (cm) | NL |
| 14 | *eps tasA*-YFP | PB178 | *epsA-O*::tet, *tasA*::kan, *amyE*::P*_hyperc1o1_*-yfp (spec) | (5) |
| 15 | *eps tasA*- mKate2 | NL111 | *epsA-O*::tet, tasA::*erm*, amyE::P*_hyperspank_*-mKate2 (cat) | NL |
| 16 | TasAop-mCherry | DR-40 | *tasA*::spec, *amyE*::tasAop-mCherry | (6) |
| 17 | P*_tapA_*-CFP | DL722 | *amyE*::P*_srfAA_*-yfp | (7)** |
| 18 | P*_srfA_*-YFP | DL823 | *lacA*::P*_tapA_*-cfp | (7)** |
| 19 | P*_tapA_*-CFP, P*_srfA_*-YFP | DL831 | *amyE*::P*_srfAA_*-yfp, *lacA*::P*_tapA_*-cfp | (7) |
| 20 | IPTG-tasA | DR6 | *tasA::km, lacA::Phyperspank-tasA* | (8) |

Source:

(1) Branda, SS, González-Pastor, JE, Ben-Yehuda, S, Losick, R & Kolter, R. Fruiting body formation in *Bacillus subtilis*. Proc Natl Acad Sci. 2001;98(20): 11621-11626.

(2) Vlamakis, H, Aguilar, C. Losick, R & Kolter, R. Control of cell fate by the formation of an architecturally complex bacterial community. Genes & Development. 2008;22: 945-953.

(3) Branda*,* SS, Chu, F, Kearns, DB, Losick, R & Kolter, R. A major protein component of the *Bacillus subtilis* biofilm matrix. Molecular Microbiology. 2006;59(4): 1229-1238.

(4) Aguilar, C., Vlamakis, H., Guzman, A., Losick, R., & Kolter, R. KinD is a checkpoint protein linking spore formation to extracellular-matrix production in *Bacillus subtilis* biofilms. MBio. 201;1(1): doi:10.1128/mBio.00035-10

(5) Beauregard, PB, Chai, Y, Vlamakis, H, Losick, R & Kolter, R. *Bacillus subtilis* biofilm induction by plant polysaccharides. Proc Natl Acad Sci. 2013;110(17): E1621-E1630.

(6) Kolodkin-Gal, I, Romero, D, Cao, S, Clardy, J, Kolter, R & Losick, R. D-Amino acids trigger biofilm disassembly. Science. 2010;328(5978):627-629.

(7) Lopez, D, Vlamakis, H, Losick, R & Kolter, R. Paracrine signaling in a bacteria. Genes & Development. 2008;23(14): 1631-1638.

(8) Romero, D, Vlamakis, H, Losick, R & Kolter, R. An accessory protein required for anchoring and assembly of amyloid fibres in *B. subtilis* biofilms. Molecular Microbiology*.* 2011;80(5): 1155-1168.

NL = Nick Lyons

* Strains are derivatives of *Bacillus subtilis* NCIB 3610

**Control: single-labelled strains that are used as control of double labeled strain, but not shown in the actual figures.
