# Supplementary material for: From Cell Differentiation to Cell Collectives: Bacillus subtilis Uses Division of Labor to Migrate
Source: PLoS Biol. 2015 Apr 20;13(4):e1002141. doi: 10.1371/journal.pbio.1002141 (PMC4403855; doi:10.1371/journal.pbio.1002141)
Supplement: S2 Table — (DOCX) [file pbio.1002141.s021.docx]

**Table S2.** Modeling parameter settings of Fig. 10 of manuscript

| Parameter | Description | Default setting | High bending rigidity | Large cell size |
| --- | --- | --- | --- | --- |
| *N* | Initial number of cells | 32 | 32 | 19 |
| *T* | Number of time steps | 17·10^6^ | 17·10^6^ | 17·10^6^ |
| *G* | Growth rate | 0.03/10^4^ | 0.03/10^4^ | 0.03/10^4^ |
| *S* | Cell size | 0.03 | 0.03 | 0.05 |
| *B* | Change in angle | 10° | 10° | 10° |
| *k* | Bending rigidity | 0.1 | 100 | 0.1 |
