# Supplementary material for: From Cell Differentiation to Cell Collectives: Bacillus subtilis Uses Division of Labor to Migrate
Source: PLoS Biol. 2015 Apr 20;13(4):e1002141. doi: 10.1371/journal.pbio.1002141 (PMC4403855; doi:10.1371/journal.pbio.1002141)
Supplement: S1 Text — (DOCX) [file pbio.1002141.s022.docx]

**Text S1. Characterization of cell types: co-expression of srfA and tapA**

The microscopy images (n = 439) of the time-course experiment (Fig. 3) were also used to examine the co-expression of *srfA* and *tapA*. This is necessary to confirm that surfactin-producing and matrix-producing cells really form mutually exclusive cell types for our growth conditions. Previous studies have shown, based on flow cytometry data, that there are no cells that strongly express both *srfA* and *tapA* [1]. This led to the conclusion that surfactin-producing and matrix-producing cells are two mutually exclusive cell types. However, when cells rarely express either *srfA* or *tapA*, one expects that finding cells that express both *srfA* and *tapA* is even less common. Not finding such cells is therefore not conclusive for answering the question of whether these cell types are mutually exclusive or not. Instead one must compare the observed co-expression pattern between *srfA* and *tapA* with the expected co-expression pattern [2]. The expected co-expression pattern follows from the assumption that the observed distributions of *srfA* and *tapA* expression are statistically independent. For example, when *srfA* is expressed at level *A* with frequency *f_A_* and *tapA* is expressed at level *B* with frequency *f_B_*, then the expected frequency of having *srfA* expressed at level *A* and *tapA* expressed at level *B* is *f_AB_*= *f_A_* · *f_B_*. S2 Fig. shows, for each combination of fluorescence intensities, whether the frequency of pixels belonging to a certain intensity combination is lower (cyan) or higher (dark blue) than that expected by chance. The combinations of fluorescence intensities for which no pixels were observed are colored grey. Interestingly, the observed co-expression pattern of *srfA* and *tapA* deviates from the expected co-expression pattern: cells that weakly express both *srfA* and *tapA* are less abundant than expected by chance (cyan area in the middle), while cells that only express either *srfA* or *tapA* are more abundant than expected by chance (dark blue area on the sides). In other words, surfactin-producing and matrix-producing cells can indeed be considered two mutually exclusive cell types. As shown in Fig. 3A, the mutually exclusive expression of *srfA* and *tapA* partly results from the temporal separation in gene expression.

References:

1. López D, Vlamakis H, Losick R, Kolter R. Paracrine signaling in a bacterium. Genes Dev. 2009;23: 1631–1638. doi:10.1101/gad.1813709

2. Van Gestel J, Vlamakis H, Kolter R. New tools for comparing microscopy images: quantitative analysis of cell types in *Bacillus subtilis*. J Bacteriol. 2015;197: 699–709. doi:10.1128/JB.02501-14
