# Supplementary material for: From Cell Differentiation to Cell Collectives: Bacillus subtilis Uses Division of Labor to Migrate
Source: PLoS Biol. 2015 Apr 20;13(4):e1002141. doi: 10.1371/journal.pbio.1002141 (PMC4403855; doi:10.1371/journal.pbio.1002141)
Supplement: S2 Text — (DOCX) [file pbio.1002141.s023.docx]

**Text S2. Quantification of van Gogh bundles**

Here we describe a quantitative method to determine if cells are part of a van Gogh bundle or not. For this purpose, we performed advanced image analysis on a number of microscopy frames that contain single cells only, van Gogh bundles or a mixture of both. All the microscopy images were taken at the colony edge, as for the images shown in Fig. 4 and Fig. 5. The images that contain single cells were acquired early in colony development, in the dendrite growth phase. The images that contain van Gogh bundles were acquired later, in the petal growth phase. The images that contain both single cells and van Gogh bundles were acquired in between both growth phases.

The images were analyzed in multiple steps (S3 Fig.). First, the microscopy images were segmented using advanced image-analysis software, called MicrobeTracker [1]. This segmentation allowed us to determine the cell outline, cell length and major axis. Cells were not treated as straight lines, but divided in similar-sized cell segments to account for the cell curvature. Second, using this output data, we could determine the spatial orientation of cells by determining the directionality of all cell segments (see segmentation in S3 Fig.). S4 Fig. shows that there is a striking difference between the spatial orientation of cells inside a population of single cells and cells within van Gogh bundles; whereas the former shows a high variability in the direction in which cells orient, the latter shows a smooth transition in which the spatial orientation of cells changes. The collection of cell segments was used for further analyses.

A number of spatial metrics could be obtained from the segmented image data. Here, we focussed particularly on the alignment of cells. For our analysis we were inspired by research on the self-organization of animal groups – i.e. fish schools and bird flocks [2]. Despite the vast differences, research on the organization of animal groups is driven by similar questions as the ones addressed in our study: how can a complex pattern of organization emerge from simple interactions between individuals? To explain the patterns that emerge in bird flocks and fish schools it has been shown that alignment of individuals is crucial. Alignment results from the attraction and repulsion of neighboring individuals, which thereby coerce each other to move in the same direction [3]. To determine the alignment of our bacterial cells, we use similar methods than those used for studying the alignment of individuals in animal groups. Namely, we randomly picked cell segments from the microscopy image, after which the average angular differences between these focal cell segments and their neighboring cell segments were determined. The neighboring cell segments were chosen within a certain radius from the focal cell segment and did not include segments from the cell to which the focal cell segment itself belongs (see S3 Fig.). Using this metric, we can determine the level of alignment in the distinct regions of a microscopy image. In case cells are perfectly aligned the average angular difference between neighboring cell segments is 0^o^. In the opposite case, when cells are oriented perpendicular with respect to each other, the average angular differences between neighboring cell segments is 90^o^. The level of alignment was determined for 10% of randomly-selected cell segments per image frame. We used a linear interpolation between these data points to determine the level of alignment over the entire image frame.

S5 Fig. shows the level of alignment across the images shown in S4 Fig. Regions with weak alignment are shown in blue and regions with strong alignment are shown in white. It is evident that the level of alignment is much higher for cells inside the van Gogh bundle than for single cells. Although regions of high alignment did occur in the single cell population, they formed isolated patches. Van Gogh bundles showed a nearly perfect alignment along the whole microscopy image, except for small regions where the bundles show strong folds. This is especially apparent when studying the vector fields – indicating the spatial orientation of cells – that are superimposed on the alignment plots in S5 Fig.

Thus, based on alignment, we can easily discriminate between images that only contain van Gogh bundles and images that only contain single cells. The question however remains how good we could discriminate between van Gogh bundles and single cells within a single microscopy image. For this purpose, we analyzed a frame that contained both single cells and van Gogh bundles, which was taken in the transition phase from dendrite growth to petal growth (corresponding to Fig. 7A in the main text). S6 Fig. shows a detailed image analysis of the mixed image frame: the spatial orientation of cells, the level of alignment and the superimposed vector field. The regions that contain van Gogh bundles clearly stand out from the alignment plot as large regions of undisrupted high levels of alignment (i.e. large white regions in the plot). In other words, the level of alignment can be used to discriminate between regions with and without van Gogh bundles within a single microscopy image.

Instead of examining the average level of alignment between a focal cell segment and its neighbors, one can also study the distribution of alignment. In other words, one can determine how the angular differences between a focal cell segment and its neighbors are distributed. As explained above, when two cells are perfectly aligned there is an angular difference of 0^o^, while in the opposite case there is an angular difference of 90^o^. S7 Fig. shows the average distribution of angular differences between neighboring cell segments. The average distribution was determined per microscopy image and, as was the case for the alignment plots in S5 Fig., was based on 10% of randomly-selected cell segments within each image. In total, four image frames were examined (S17 Fig.). Two of these images correspond to those analyzed in S4 Fig. and S5 Fig. The other two were newly analyzed images and used as replicates: one of these new images only contained van Gogh bundles and the other one only single cells. Thus, the red distributions in S7 Fig. correspond to the two microscopy images with van Gogh bundles and the blue distributions correspond to the two microscopy images with only single cells. As expected, the distributions corresponding to the van Gogh bundles were skewed towards the left in comparison to those corresponding to single cells, meaning that neighboring cell segments showed smaller angular differences (i.e. stronger alignment). Interestingly, the two images with van Gogh bundles showed nearly identical distributions of angular differences. The same was observed for the other two distributions. Thus, the distribution of alignment is very consistent between image frames and can therefore be used as a reliable indicator for absence or presence of van Gogh bundles.

Given the large database of segmented cells, we could also examine the average characteristics of cells that occur inside a van Gogh bundle and cells that are part of a single cell population. We determined the cell length and curvature based on phase-contrast images. The inset of S7 Fig. shows the average cell shapes. Cells in van Gogh bundles appear longer than their single cells siblings. The curvature of the cells is more-or-less the same, although cells inside the van Gogh bundles appear more curved because they are longer.

All in all, van Gogh bundles can accurately be quantified based on the alignment of cells. Although, the level of alignment is a powerful metric, additional metrics are required for an even more comprehensive view on van Gogh bundles. For example, one could also determine the width and length of the van Gogh bundles. Such metrics require a substantial larger amount of image acquisition and analyses, but would be interesting to pursue in future studies.

References:

1. Sliusarenko O, Heinritz J, Emonet T, Jacobs-Wagner C. High-throughput, subpixel precision analysis of bacterial morphogenesis and intracellular spatio-temporal dynamics. Mol Microbiol. 2011;80: 612–627. doi:10.1111/j.1365-2958.2011.07579.x

2. Hemelrijk CK, Hildenbrandt H. Schools of fish and flocks of birds: their shape and internal structure by self-organization. Interface Focus. 2012;2: 726–737. doi:10.1098/rsfs.2012.0025

3. Katz Y, Tunstrøm K, Ioannou CC, Huepe C, Couzin ID. Inferring the structure and dynamics of interactions in schooling fish. Proc Natl Acad Sci. 2011;108: 18720–18725. doi:10.1073/pnas.1107583108
