# Supplementary material for: From Cell Differentiation to Cell Collectives: Bacillus subtilis Uses Division of Labor to Migrate
Source: PLoS Biol. 2015 Apr 20;13(4):e1002141. doi: 10.1371/journal.pbio.1002141 (PMC4403855; doi:10.1371/journal.pbio.1002141)
Supplement: S3 Text — (DOCX) [file pbio.1002141.s024.docx]

**Text S3. TasA distribution**

TasA is produced by matrix-producing cells, which predominantly occur inside the van Gogh bundles. In this section, we examine the spatial distribution of TasA using fluorescence microscopy images of a TasA-mCherry strain (i.e. the TasA protein is fused with a red fluorescent protein). Three questions were addressed:

(1) Does TasA diffuse outside the van Gogh bundles?

(2) Does TasA preferentially localize to the pole-to-pole interactions between cells?

(3) Does TasA localize to the pole-to-pole interactions between *tasA* mutants inside the van Gogh bundle?

*Only a small fraction of TasA diffuses towards the single cells that surround the van Gogh bundle.*

To determine if TasA strictly occurs in the van Gogh bundles, where it is produced, or also diffuses to surrounding cells, we performed a detailed analysis on a section of the microscopy image of Fig. 7A (see inset of S9 Fig.). The image section was selected such that the left side consisted of van Gogh bundle and the right side of single cells, as also apparent from the level of alignment between cells (S9 Fig., blue line).

S9 Fig. shows the TasA distribution for a horizontal cross-section of the image section. The fluorescence intensity was normalized, such that the background expression is equal to 0 and the highest observed fluorescence value is equal to 1. As expected, based on the visual examination of the fluorescence image (Fig. 7A), TasA is predominantly localized to the van Gogh bundles. The sharp peaks correspond to the pole-to-pole interactions between cells in the van Gogh bundle, which will be analyzed in detail in the next section. Interestingly, a fraction of TasA did diffuse to the surrounding single cells, although this fraction is only marginal in comparison to the fluorescence peaks observed in the van Gogh bundles.

*TasA predominantly localizes to the pole-to-pole interactions between cells.*

From the previous analysis and the visual inspection of the fluorescence images of Fig. 7A, one would conclude that TasA preferentially localizes to the pole-to-pole interactions between cells in the van Gogh bundle. Here, we perform a quantitative image analysis, to confirm if TasA is indeed localized to the pole-to-pole interaction points between cells.

Given the strong alignment of cells inside the van Gogh bundles, there are only two types of cell-to-cell interactions: pole-to-pole and side-to-side cellular interactions (only non-aligned cells can have pole-to-side interactions). To determine to which of these cell-to-cell interactions TasA predominantly localizes, we analyzed the fluorescence intensity along hundreds of line segments. We examined two types of line segments (see S10 Fig.): line segments along a cell’s major axis at the cell poles (red; aimed to examine the pole-to-pole interactions) and line segments along a cell’s minor axis at the cell sides (blue; aimed to examine the side-to-side interactions). S10 Fig. shows the fluorescence intensity along each of the examined line segments as well as the average gradient in fluorescence intensity. As expected, on average there were much higher concentrations of TasA at the pole-to-pole interactions between cells (red) than at the side-to-side interactions between cells (blue). This shows that TasA predominantly localized to the cell poles. One should note that TasA also accumulated at ‘loose’ pole ends, where no neighboring cells are present, so the accumulation of TasA does not necessarily require two interacting cells (S9 Fig. and S10 Fig.). Yet, such ‘loose’ poles are relatively rare inside the van Gogh bundles, since cells form chains.

*TasA does not localize to the pole-to-pole interactions between* tasA *mutant cells.*

As described above, a part of TasA produced by cells inside the van Gogh bundles diffuses to the single cells surrounding the bundle. However, despite the presence of TasA, we did not observe an accumulation of TasA around the poles of cells outside the van Gogh bundles. It can be that cells outside the van Gogh bundles do not express the necessary proteins to sequester TasA. It has been shown that the assembly of TasA into amyloid-like fibers and the binding of these fibers on the cell wall depends on TapA, an accessory protein that forms discrete foci in the cell envelope [1,2]. When cells do not express TapA the allocation of TasA towards the cell poles might be hampered.

We therefore examined if TasA could localize to the pole-to-pole interactions between *tasA* mutants that are part of a van Gogh bundle. We examined van Gogh bundles that consist of two strains: a WT strain that produces the fusion TasA-mCherry and a *tasA* mutant strain. The strains occur side-by-side as cell chains in the chimeric van Gogh bundles. As *tasA* mutant cells are part of the van Gogh bundle, they are expected to express the proteins necessary to sequester TasA. We examined the TasA concentration along line segments at the pole-to-pole interactions between either WT cells (red, S11 Fig.) or *tasA* mutant cells (blue, S11 Fig.). S11 Fig. shows the fluorescence intensity along each of the line segments as well as the average. As is the case for the single cells surrounding the van Gogh bundles, a fraction of TasA diffused from the TasA-producing cells to the *tasA* mutant cells within the van Gogh bundles. Yet, interestingly, there was no preferential allocation of TasA towards the pole-to-pole interactions between *tasA* mutant cells, while there was such allocation between WT cells. Thus, even though a fraction of TasA diffuses away from the cell, it does not localize to the pole-to-pole interactions between cells that do not produce TasA themselves. It seems that a substantial fraction of the TasA that is produced by a WT cell is sequestered towards its own poles.

References:

1. Romero D, Aguilar C, Losick R, Kolter R. Amyloid fibers provide structural integrity to *Bacillus subtilis* biofilms. Proc Natl Acad Sci. 2010;107: 2230–2234. doi:10.1073/pnas.0910560107

2. Romero D, Vlamakis H, Losick R, Kolter R. An accessory protein required for anchoring and assembly of amyloid fibres in *B. subtilis* biofilms. Mol Microbiol. 2011;80: 1155–1168. doi:10.1111/j.1365-2958.2011.07653.x
